# In Situ Generation by Cyclization of an Organic Structure Directing Agent for the Synthesis of High Silica Zeolite ERS‐7

**DOI:** 10.1002/chem.202500327

**Published:** 2025-05-07

**Authors:** Magdalena M. Lozinska, Ruxandra G. Chitac, Elliott L. Bruce, Malavika Manoj, Yuanyuan Du, Daniel M. Dawson, Sharon E. Ashbrook, Paul A. Cox, Paul A. Wright

**Affiliations:** ^1^ EaStCHEM School of Chemistry University of St Andrews Purdie Building, North Haugh St Andrews KY16 9ST UK; ^2^ Orbital Materials 11 Deerpark Drive, Monmouth Junction New Jersey 08852 USA; ^3^ Institute of Chemical Science School of Engineering and Physical Sciences, Heriot‐Watt University Edinburgh EH14 4AS UK; ^4^ Centre of Magnetic Resonance EaStCHEM School of Chemistry University of St Andrews St Andrews UK; ^5^ School of Pharmacy and Biomedical Sciences University of Portsmouth Portsmouth PO1 2DT UK

**Keywords:** cationic polymer, ESV topology type, in situ OSDA formation, zeolite synthesis

## Abstract

Zeolites with high framework Si/Al ratios are of interest for industrial applications due to their hydrothermal stability. They are usually synthesized in the presence of pre‐prepared organic structure directing agents (OSDAs). The high silica ERS‐7 zeolite (topology type **ESV**) can be crystallized using *N*,*N*‐dimethylpyrrolidinium (dmpyrr) that is formed in situ via cyclization of *N*,*N*,*N’*,*N’*‐tetramethyl‐1,4‐diaminobutane (tmdab) when a cationic polymer is also present. The in situ generation of dmpyrr is demonstrated by solid‐state ^13^C NMR spectroscopy and supported by both chemical analyses and comparative syntheses using pre‐prepared dmpyrr. The cationic polymer inhibits the crystallization of mordenite, which is otherwise observed to be the favored product. The ERS‐7 prepared via in situ dmpyrr synthesis (Si/Al = 14) is characterized by PXRD and solid‐state NMR spectroscopy. The CO_2_ adsorption seen for the H‐ and Na‐forms indicates interactions with accessible Na^+^ cations. The synthetic studies indicate the potential for in situ generation of OSDAs to reduce the need for extended OSDA syntheses.

## Graphical abstract

A synthetic route is established to high silica zeolite ERS‐7 templated by a dimethylpyrrolidinium organic structure directing agent (OSDA) in the presence of a cationic polymer, both formed in situ from organic precursors. The polymer enables the cyclization that gives the OSDA and inhibits the crystallization of the competing zeolite mordenite.

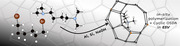

## 1 Introduction

Zeolites find widespread application in adsorption and catalytic processes so understanding and modifying their hydrothermal synthesis to give materials with new structures and compositions is of continuing interest.^[^ ^1^, ^2^ ^]^ The use of organic structure directing agents (OSDAs), particularly organic cations, to template zeolite structures is a well‐established route to controlling product structure type. This results from framework stabilization by matching the pore structure with the OSDA shape, and so optimizing framework‐OSDA non‐bonding interactions.^[^ ^3^, ^4^ ^]^ It also leads to the preparation of high silica zeolites, because the low charge density of organic alkylammonium cations is matched by the low overall charge density (and Al content) of the frameworks that form around them. The Si/Al ratio of zeolites is an important parameter in determining their properties, such as cation exchange capacity, acid concentration and strength, and stability. Higher Si/Al ratios impart enhanced hydrothermal stability to zeolites, particularly in the acid form.^[^ ^5^ ^]^ High silica zeolites can in some cases be difficult to crystallize and require the use of fluoride media and seeds, so fluoride‐ and seed‐free routes are attractive to reduce hazards and cost.^[^ ^6^ ^]^

OSDAs are typically alkylammonium cations, prepared in advance of the hydrothermal zeolite synthesis through single or multiple organic reactions, ranging from simple alkylations to additions and cyclizations.^[^ ^7^, ^8^, ^9^ ^]^ These routes have associated separation and purification steps, leading to additional experimental effort and expense. In this regard, it is interesting to note that in some cases it is possible to prepare OSDAs within the hydrothermal zeolite crystallization step by adding the required precursors to the gel, which subsequently react to form an OSDA that directs crystallisations. In the example given by Bats et al., following on from a patent,^[^ ^10^, ^11^ ^]^ zeolite EU‐1 was prepared by adding the precursors of its hexamethonium OSDA (dibromohexane and trimethylamine) to the gel before hydrothermal treatment. Solid state NMR showed the hexamethonium template had been prepared in situ and was incorporated in the zeolite product. This approach simplifies the synthetic method and reduces the use of energy and solvent, and so accords with the principles of green chemistry proposed for zeolite synthesis.^[^ ^12^ ^]^

**Scheme 1:**
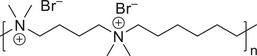
Cationic polymer **1**, polydimethylammoniumbutane, dimethylammoniumhexane bromide.

Among the many different OSDAs that have been explored, cationic organic polymers have been explored as templates for the synthesis of a number of zeolite structure types. Their structure‐directing action is usually confirmed by solid state ^13^C NMR of the as‐prepared solids that confirm they remain in the zeolite pores after crystallization. One of the first examples was the synthesis of fault‐free gmelinite using the cationic polymethylene‐DABCO polymer^[^ ^13^ ^]^ which templates 1D twelve‐membered ring (12R) channels. This ensures there are no stacking faults of the type that can arise when using small molecule OSDAs, which lead to pore restriction. Similar polymethylene‐DABCO cationic polymers act to template the zeotype metalloaluminophosphate MgAPO‐31, which also possesses 1‐dimensional 12R channels.^[^ ^14^ ^]^ Other cationic polymers have been shown to act as OSDAs. ZSM‐23 and ZSM‐12, high silica zeolites with 1D channels bound by 10Rs or 12Rs, respectively, have been templated by more complex polymers.^[^ ^15^, ^16^ ^]^ Molecular modeling indicates a close correlation between repeat units of the polymers and of the channel wall of the zeolite frameworks. Additionally, the use of polyquaternary templates has a strong effect on zeolite morphology. While a polymeric cation of the form [‐N^+^(CH_2_CH_2_CH_3_)_2_‐(CH_2_)_6_‐]*_n_*, described as a “bond‐linked” polymeric analog of tetrapropylammonium,^[^ ^17^ ^]^ is found to act as an OSDA for ZSM‐5 nanosheets, a polyquaternary template [‐N^+^(CH_3_)_2_‐(CH_2_)_6_‐N^+^(CH_3_)_2_‐(CH_2_)_5_‐]_n_ gives nanosheets of zeolite Beta.^[^ ^18^ ^]^ Recently, the synthesis of an aluminosilicate version of ITQ‐13 (topology type **ITH**) has been realised using the cationic poly[dimethylammoniumbutane, dimethylammoniumhexane] polymer (Scheme 1) in the presence of fluoride mineraliser.^[^ ^19^ ^]^

The readily‐available cationic polymer polydiallyldimethyl‐ammonium chloride PDADMAC has also been widely investigated as an additive in zeolite syntheses. It has been found to act as an OSDA for zeolite Beta, and also to introduce mesopores in that 3D 12R zeolite, leading to hierarchical meso‐microporosity.^[^ ^20^ ^]^ Other reports show that PDADMAC has important but indirect effects in controlling nucleation and crystallization of low silica zeolites such as zeolite Rho and EMT without becoming incorporated at a significant level.^[^ ^21^, ^22^ ^]^ More recently, Rimer et al. observed that the addition of low concentrations of some cationic polymers accelerates the crystallization of chabazite (SSZ‐13, topology type **CHA**) while not becoming incorporated in the pores themselves.^[^ ^23^ ^]^ Such effects are thought to result from polymer‐induced particle agglomeration in the pre‐nucleation stage.^[^ ^24^ ^]^

While investigating the in situ generation of OSDAs during hydrothermal zeolite synthesis, we examined the effect of polymeric cations such as **1** (Scheme 1) with sodium aluminosilicate gels under conditions that were known to prepare high silica zeolite structure types such as MCM‐22(P), TNU‐9 and EU‐1.^[^ ^25^ ^]^ Polymer **1** was either prepared within the hydrothermal syntheses from added reagents or before the zeolite synthesis and then added to the gel prior to heating. Under some conditions, we observed the crystallization of a high silica form of ERS‐7 zeolite (topology type **ESV**).^[^ ^26^ ^]^

**Figure 1:**
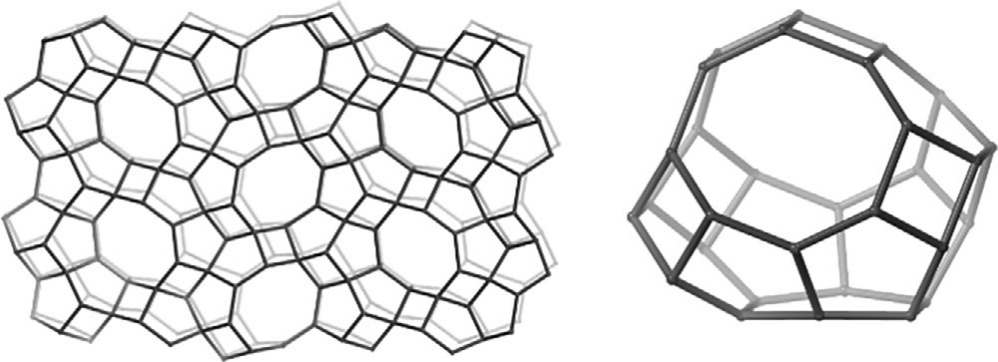
(Left) The **ESV** framework, represented by T‐T links, where T represents central atoms of TO_4_ tetrahedra, viewed along the [010] direction. (Right) A 17‐hedral *esv* “picnic basket” cavity in **ESV**, again represented by T‐T links.

ERS‐7 has one‐dimensional zig‐zag channels resulting from 17‐hedral (“picnic basket”‐ shaped, *esv*) cavities which each share 8R (8‐ring) windows with two adjacent, identical cavities (Figure 1). The framework itself comprises chains of smaller 11‐hedral (*mim*) cages that share 6Rs, each chain of cages being connected to neighbors by single TOT linkages and 4Rs. Campbell et al. prepared ERS‐7 with Si/Al = 8 using *N*,*N*‐dimethylpiperidium (dmpip) as the OSDA.^[^ ^27^, ^28^ ^]^ Subsequently, it has been synthesized by Chevron (as SSZ‐102) using dimethyl‐DABCO^[^ ^29^ ^]^ and by Bae and Hong in a high silica form (Si/Al = 13.5) via a fluoride‐mediated synthesis using choline.^[^ ^30^ ^]^ Within these literature reports, ERS‐7 has been shown to display selective uptake of CO_2_ which is expected because small pore zeolites display attractive properties for CO_2_ adsorption for air purification and carbon capture.^[^ ^31^, ^32^, ^33^ ^]^

Here, we have investigated our fluoride‐free route to a high silica ERS‐7 and the role of organic additives in the crystallization. The organic template is found to form by the reaction of the organic precursors via an unexpected mechanism: as well as leading to a new template for ERS‐7, this observation suggests an approach for in situ OSDA synthesis during zeolite crystallization that is distinct from the work described by Bats et al.^[^ ^10^ ^]^ In parallel, cationic polymeric species act to control nucleation of ERS‐7 and inhibit competing phases and are suggested to act as methylating agents. These results suggest an attractive alternative route for the crystallization of aluminosilicate zeolites.

## 2 Results and Discussion

Initial syntheses were performed by adding the cationic polymer **1** or its precursor molecules, *N,N,N′,N′‐*tetramethyl‐1,4‐diaminobutane (tmdab) and 1,6‐dibromohexane, to a sodium aluminosilicate gel (Table 1) under conditions known to be suitable for the crystallization of high silica zeolites in the presence of cationic OSDAs.^[^ ^25^ ^]^ In the absence of added organics, zeolite mordenite (framework topology **MOR**) crystallized.

**Table 1:** Gel compositions (as molar ratios) that give ERS‐7 (**ESV**) zeolite products. All gels were aged for 24 h under static conditions, followed by crystallization under rotation at 160 °C for 7 days. Abbreviations: diBrC6 = 1,6‐dibromohexane, tmdab = *N,N,N',N'*‐tetramethyl‐1,4‐diaminobutane, dmpyrr = dimethylpyrrolidinium bromide, poly = polymer **1** (monomer unit [N(CH_3_)_2_C_4_H_8_N(CH_3_)_2_C_6_H_12._Br_2_]).

Experiment No. Organic additives	Na: Al: Si	diBrC6	tmdab	dmpyrr	poly	H_2_O
E1 Polymer precursors	22: 1: 30	6	6‐7	0	0	1335
E2 Polymer + tmdab	22: 1: 30	0	1	0	6	1335
E3 Polymer + dmpyrr	24: 1: 30	0	0	3	3	1183
E4 Low polymer + dmpyrr	23: 1: 30	0	0	3	0.5	1179
E5 dmpyrr^*^	22: 1.5: 30	0	0	4.5	0	560

* seeded with ERS‐7 crystals

**Figure 2:**
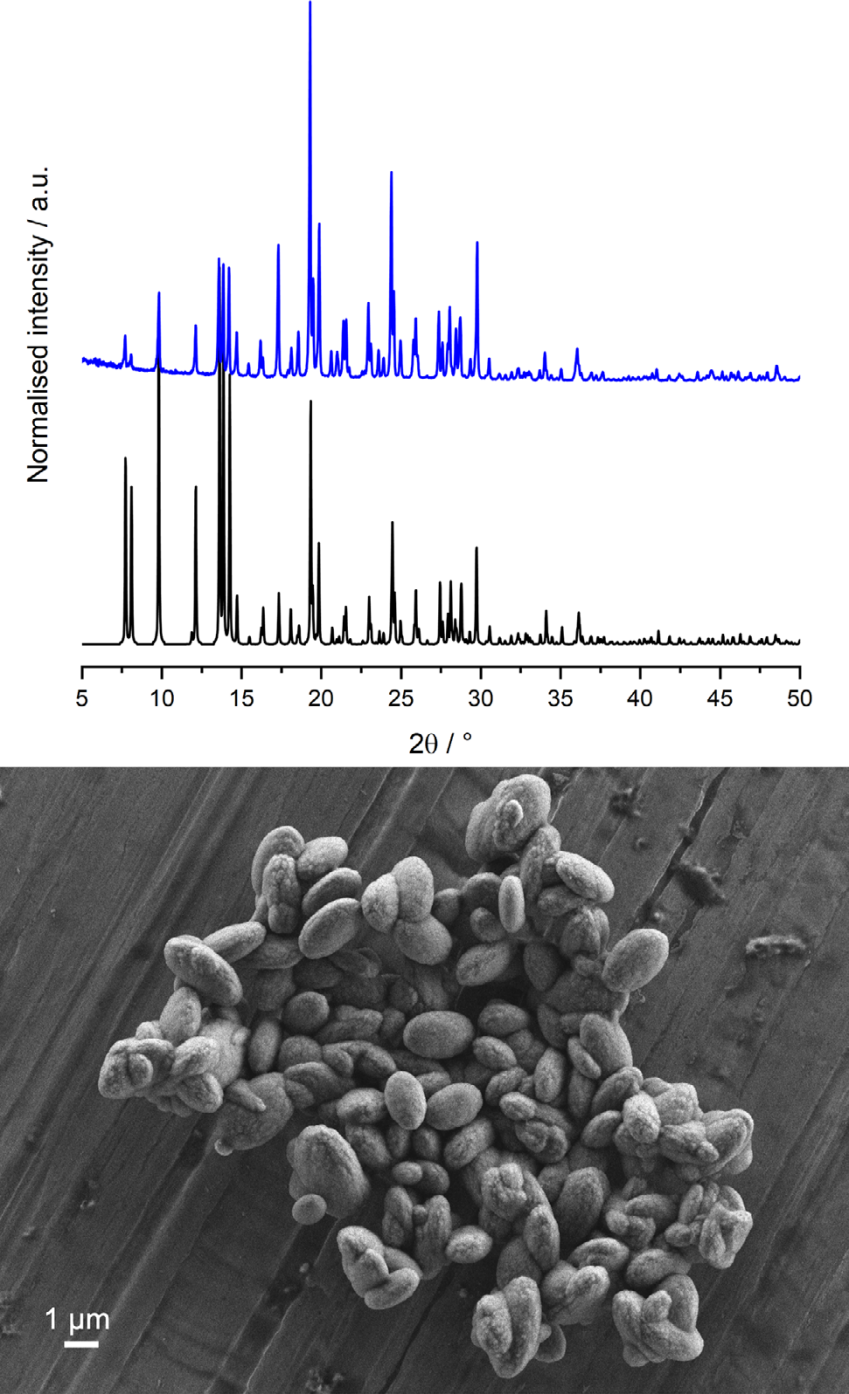
(Above) PXRD pattern of as‐prepared ERS‐7 (Cu K_α1_, 1.54056 Å) synthesized using an excess of tmdab in a tmdab+diBrC6 prep (in a 7:6 ratio) (top) compared with that simulated from the framework structure available from IZA database^[^ ^26^ ^]^ (bottom). Differences in intensity result from the presence of OSDA in the as‐prepared material. (Below) SEM image of ERS‐7 prepared with tmdab and diBrC6.

Although no crystallization was observed using pre‐made **1** as the only organic additive, indicating the polymer acts to inhibit mordenite crystallization, zeolite ERS‐7 was sometimes observed as the crystalline product when adding the polymer precursors in the ratio 1:1 (Experiment E1, Table 1). It was subsequently found that ERS‐7 was reliably crystallized if an excess of *N,N,N′,N′‐*tetramethyl‐1,4‐diaminobutane (tmdab) was added (diamine: dibromoalkane = 7: 6, E1) in the in situ polymer preparation synthesis (see Figure 2 for powder X‐ray diffraction (PXRD) and scanning electron microscopy (SEM) images of ellipsoidal crystals that are 2–3 µm in length). Similarly, ERS‐7 crystallized if the equivalent excess of tmdab was added to preparations that included the pre‐made cationic polymer **1** (E2, Table 1). In these last cases, reducing the amount of polymer in the preparation to one‐half of the initial value, but keeping the amount of added tmdab constant resulted in the co‐crystallisation of mordenite with poorly crystalline ERS‐7 (see Figure S2). However, using only the equivalent excess of tmdab, with no polymer, gives mordenite (Figure S2). Adding *N*,*N*,*N’*,*N’*‐tetramethyl‐1,6‐diaminohexane in place of tmdab in analogous preparations did not give any crystalline product (Table S2 and Figure S3).

These results showed both tmdab and polymer **1** were required to prepare ERS‐7 zeolite reliably. We hypothesized that the polymer inhibits the crystallization of mordenite, which otherwise forms very readily in this system without any organic additives, while the tmdab was involved in templating the ERS‐7.

**Figure 3:**
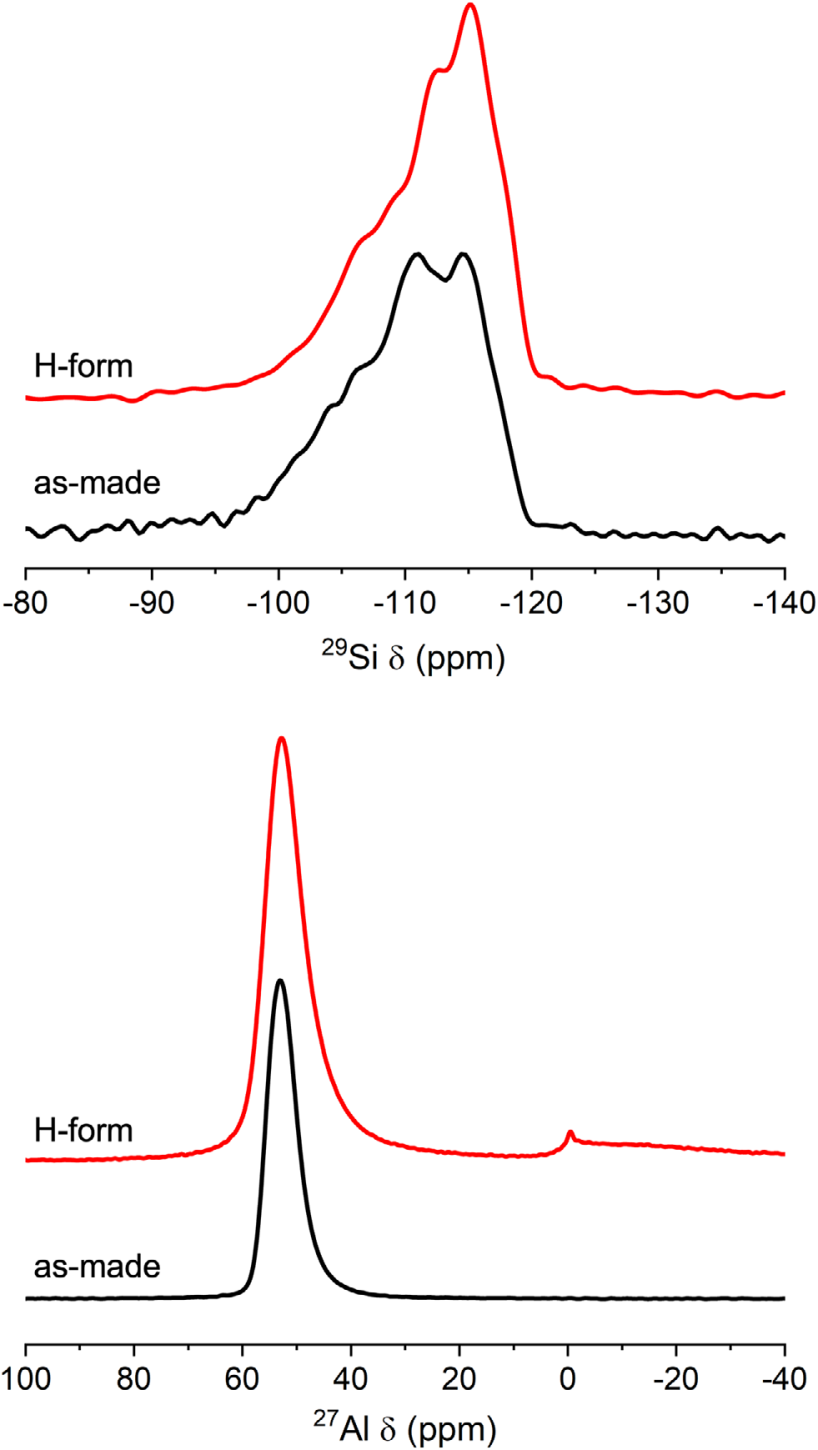
(Above) ^29^Si (9.4 T, 10 kHz) and (below) ^27^Al (9.4 T, 14 kHz) MAS NMR spectra of ERS‐7. Black: spectra from as‐prepared form, red: spectra from hydrated H‐form.

The Si/Al ratio of ERS‐7 produced via this route was determined as 14.3 using SEM‐EDS (energy dispersive X‐ray spectroscopy). The ^29^Si MAS NMR spectrum of the as‐prepared material shows two peaks at −115 and −111 ppm, along with a shoulder at ca. −106 ppm (Figure 3), which are similar to the signals observed in other NMR spectra of ERS‐7 reported in the literature.^[^ ^30^ ^]^ ERS‐7 possesses six crystallographically different T sites in the *Pnma* space group, so an accurate deconvolution of the peaks in the ^29^Si NMR spectrum is challenging and beyond the scope of this paper. The ^27^Al MAS NMR spectrum showed a single peak centered at 50 ppm corresponding to tetrahedral framework aluminium (Figure 3), with a tail to the low‐frequency characteristic of disorder in the framework.

Calcination, ion exchange to the NH_4_‐form, and deammoniation produced a highly crystalline H‐ERS‐7 with a micropore volume of 0.08 cm^3^ g^−1^ (Figures S4, S5). The ^29^Si MAS NMR spectrum of H‐ERS‐7 showed some changes in intensity and shifts, with maximum intensity at −115 ppm. Only a very small amount of octahedrally‐coordinated aluminum (at 0 ppm) was observed in the ^27^Al MAS NMR spectrum (Figure 3). The NMR spectra and N_2_ adsorption isotherm were similar to those reported for high silica ERS‐7.^[^ ^30^ ^]^

Bae and Hong report the sodium form of their high silica ERS‐7 possesses reasonable CO_2_ uptake (2.7 mmol g^−1^ at 298 K and 1 bar) and good CO_2_/CH_4_ selectivity (12, at 0.5 bar and 298 K). To confirm that our high silica ERS‐7 has similar CO_2_ adsorption properties, its fully Na‐exchanged form was prepared (see Experimental Section). EDS gave a unit cell composition of Na_3.3_Al_3.3_Si_44.8_O_96_ for the dehydrated form.

**Figure 4:**
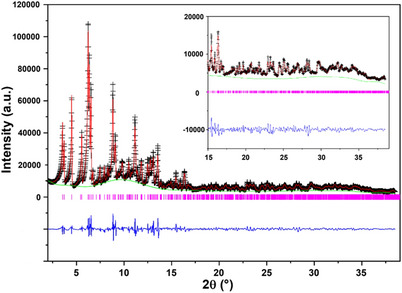
Rietveld plot of PXRD data (Mo K_α1_, λ = 0.70926 Å) of dehydrated Na‐ERS‐7.

**Figure 5:**
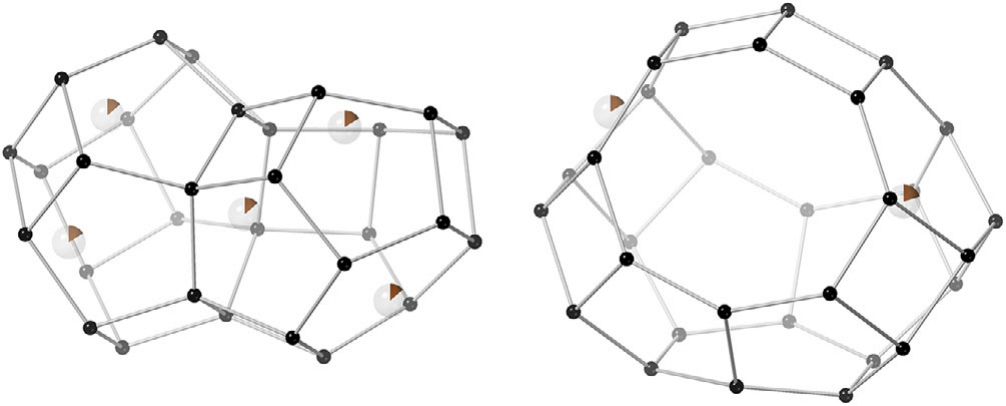
Positions of Na^+^ cations in dehydrated Na‐ERS‐7, found through Rietveld refinement of PXRD data. (Na – brown with part‐filled circles matching the partial occupancies of the sites, silicon – black, O sites omitted for simplicity).

**Figure 6:**
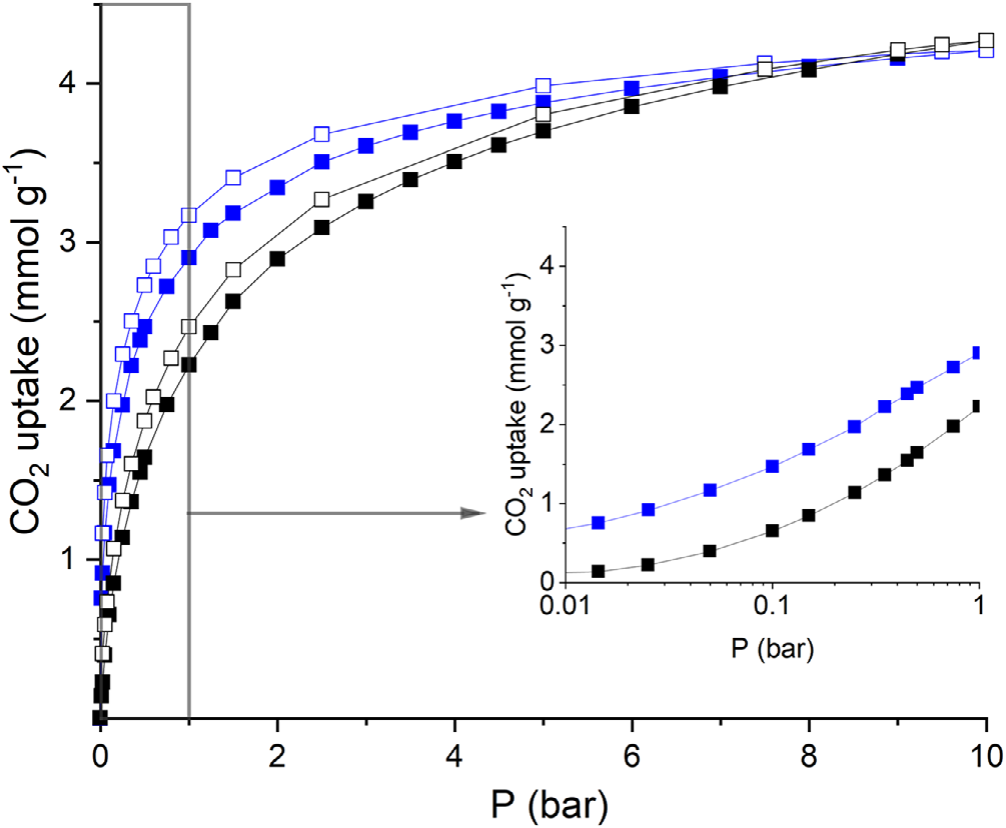
Plots showing CO_2_ sorption at 298 K, up to (left) 10 bar and (right, on log scale) 1 bar, on H‐ERS‐7 (black) and Na‐ERS‐7 (blue). Adsorption: closed symbols; desorption: open symbols.

The structure of the dehydrated Na‐form was determined via Rietveld refinement^[^ ^34^ ^]^ against PXRD data. (See Figure 4 and Tables S2 and S3 for details). Na^+^ cations in the structure are located in three sites (Figure 5). In addition to the two Na^+^ cation sites found in the 6Rs of the smaller *mim* cages, a third Na^+^ site was found in the single 6Rs of the *esv* cavities where Na^+^ cations are accessible to CO_2_ molecules: all three sites had fractional occupancy of *ca* 0.17. CO_2_ uptake at 298 K on the Na‐ERS‐7 was compared with that of H‐ERS‐7 (Figure 6). As expected, the Na‐form possessed a steeper isotherm, due to the stronger interaction of CO_2_ with the Na^+^ cations within the larger cavities than with the protons of the H‐form, with uptake at 1 bar of ca. 2.8 mmol g^−1^ (very close to that recorded by Bae and Hong at the same conditions).

These data confirm that a high silica version of ERS‐7 has been prepared without the use of fluoride ions for the first time. It was therefore of importance, as well as of fundamental interest, to understand the action of the organic additives in this synthesis.

### 2.1 Understanding the Role of the Diamine as an OSDA

**Figure 7:**
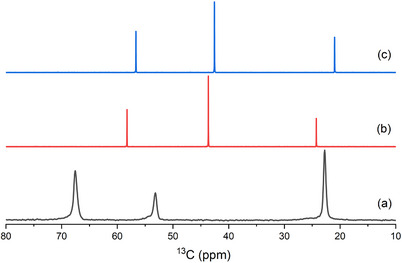
a) ^13^C (9.4 T, 12.5 kHz) solid‐state CP MAS NMR spectrum of ERS‐7, crystallized from a gel containing tmdab and 1,6‐dibromohexane as organic additives, compared with solution‐phase ^13^C NMR spectra of tmdab in b) unprotonated and c) protonated forms.

Considering the synthetic results described above and given in Table 1, both tmdab and the cationic polymer play important roles in the synthesis, as discussed below. The ^13^C CP MAS NMR spectra of the as‐prepared ERS‐7 showed three well‐resolved peaks at 22.8, 53.2, and 67.6 ppm (Figure 7) while the ^15^N MAS NMR spectrum shows only one sharp peak, at −313.4 ppm, typical of a quaternary ammonium species (Figure S6).

The ^13^C MAS NMR spectra indicated that any polymer synthesized in situ was not included in the ERS‐7 structure, as this would lead to additional resonances (see Section S1 of the SI for the solution‐phase ^13^C NMR spectra of the polymer). While the three ^13^C resonances seen might suggest the inclusion of the intact tmdab, comparison with solution‐phase ^13^C NMR spectra of the diamine, either neutral or protonated (Figure 7), shows signals at quite different chemical shifts compared to those observed in as‐prepared ERS‐7.

Further evidence also argues against the direct incorporation of tmdab, most notably elemental analysis, which indicates an organic (CHN) content of *ca*. 12 wt.% with a C/N molar ratio of 6.1, rather than the expected ratio of 4 for tmdab (observed, C 8.55 wt.%, N 1.63 wt.%). The observed C/N molar ratio and the three distinct signals in the NMR spectrum are consistent with an alkylammonium OSDA containing one N atom.

**Figure 8:**
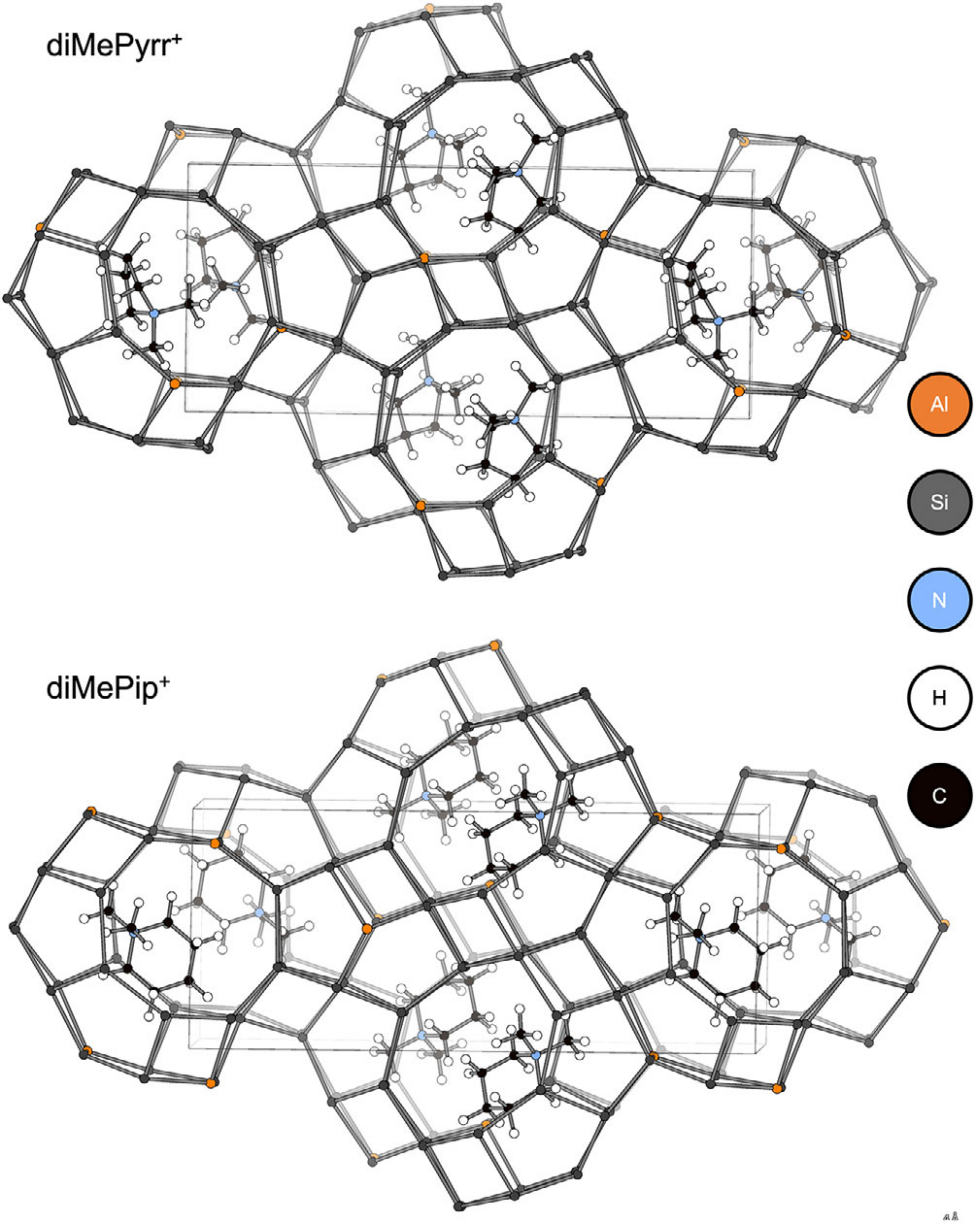
Computationally‐simulated energy minimum positions of (top) *N,N‐*dimethylpyrrolidinium cations and (bottom) *N,N‐*dimethylpiperidinium cations in the picnic‐basket *esv* cavities of the ESV framework represented with T‐T connection.

Additionally, TGA (Figure S7) suggests that if tmdab molecules were incorporated, it would be at a level of 3.3 per unit cell, so that >3 out of the 4 “picnic basket” cavities in one unit cell would each be filled by one molecule. However, computational modeling of the lowest energy configuration of tmdab in ERS‐7 suggests it would stretch over two adjacent cavities, in which case the maximum occupancy would be two per unit cell (Figure S8). Associated Rietveld refinement against the PXRD data of as‐prepared ERS‐7 gave a relatively poor fit to the data using this model and restricted the level of occupancy (Figure S9, *R*_wp_ = 19.8%). One alternative hypothesis we examined was that the tmdab could adopt a cyclic, intramolecularly H‐bonded, monoprotonated species, as observed under very different conditions,^[^ ^35^ ^]^ but this was not supported by computational modeling of the species within the *esv* cavity,^[^ ^36^, ^37^ ^]^ because unfavorable “close” contacts between molecule and cavity were observed (Figure S8). Nevertheless, this model did give a better fit to the PXRD data (*R*_wp_ = 14.9%). According to these considerations, the suggestion of any alternative OSDA to tmdab must be able to explain the analytical and spectroscopic data. Hints from the literature pointed us in the direction of a cyclic species as a candidate. The first literature report of the synthesis of ERS‐7 was achieved by the cyclic dimethylpiperidinium cation,^[^ ^27^ ^]^ which our modeling showed to be a good fit to the *esv* cavity (Figure 8). Furthermore, catalyzed cyclizations of α,ω‐diamines to give cyclic species such as pyrrolidine, have been reported previously in aqueous systems.^[^ ^38^ ^]^ If such a reaction were to occur with the tmdab it would form a pyrrolidinium ring, and NMR spectra of dimethylpyrrolidinium formed within pores in reactions over ZSM‐5^[^ ^39^ ^]^ gave solid‐state NMR ^13^C shifts similar to those we observed in our as‐prepared ERS‐7 (reported δ^13^C: 23.0 ppm, 52.5 ppm, 64.4 ppm; observed δ^13^C: 22.8 ppm, 53.2 ppm, 67.6 ppm).

Therefore, we hypothesized that *N*,*N*‐dimethylpyrrolidinium (dmpyrr) was the OSDA for ERS‐7 in our experiments. Computational modeling of the OSDA binding energy in aluminosilicate **ESV** structures using DFT performed with Materials Studio and the Machine‐learned forcefield Orb V2^[^ ^40^, ^41^ ^]^ (see Experimental Section for simulation methodology) suggested that an optimized location within the cavity gave favorable nonbonding interaction energies and no undesirable close contacts (Figure 8 and Table S7). The interaction energy and the fit of dimethypyrrolidinium was similar to the one simulated for the literature‐reported OSDA, dimethylpiperidinium (dmpip) cation (Figure 8, bottom, Table S7).

**Figure 9:**
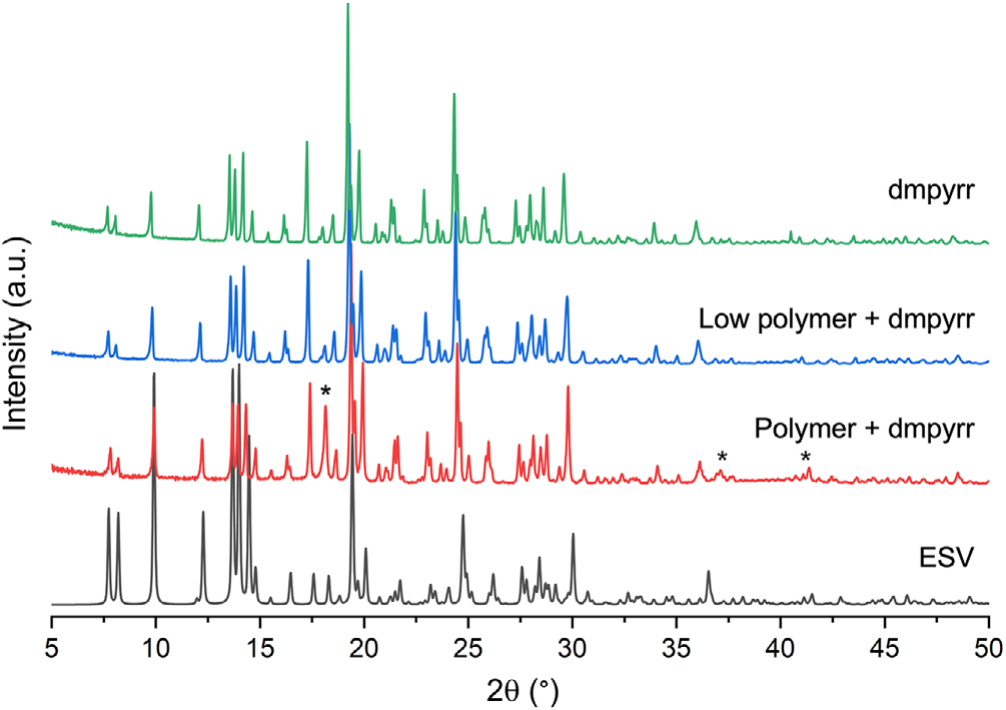
PXRD patterns of as‐prepared ERS‐7 samples (Cu K_α1_, 1.54056 Å) synthesized with added dimethypyrrolidinium bromide in the presence of varying amounts of cationic polymer (see Table 1 for details). The patterns are compared with the simulated ESV pattern. Asterisks indicate peaks arising from the Teflon support used when running the flat plate measurement.

**Figure 10:**
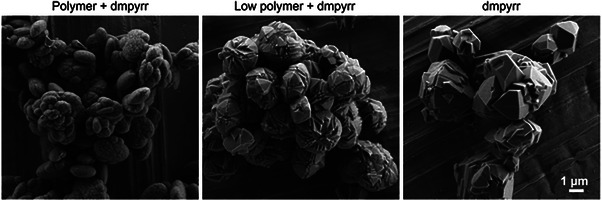
SEM images of the ERS‐7 materials synthesized with dmpyrr and varying amounts of cationic polymer (see Table 1 for details).

Experimentally, we investigated the feasibility of dmpyrr as an OSDA by adding pre‐synthesized dimethylpyrrolidinium bromide (prepared in‐house, details in section S1 of SI) to a synthesis gel containing cationic polymer at the levels of the original synthesis (Table 1). PXRD indicated highly crystalline and phase‐pure ERS‐7 had crystallized (Figure 9), which possessed oval‐pill‐shaped morphology (Figure 10).

To determine whether the cationic polymer was required if pre‐prepared dmpyrr was present, the polymer was added at reduced amounts down to zero, and some small adjustments were made to the gel composition (Table 1). The results of these syntheses were to prepare highly crystalline ERS‐7 materials down to a “low” polymer level ((C_14_N_2_H_32_)^2+^‐repeat unit)/SiO_2_ = 0.02), below which an inorganic‐only mordenite crystallized (EDS for the mordenite indicated Na/Al = 0.9; PXRD and SEM images in Figure S11). SEM indicates an increase in the crystal size of ERS‐7 as the amount of polymer is reduced. EDS indicates Si/Al ratios of the ERS‐7 solids to be in the range 10.5‐11.5, slightly lower than when using the tmdab.

**Figure 11:**
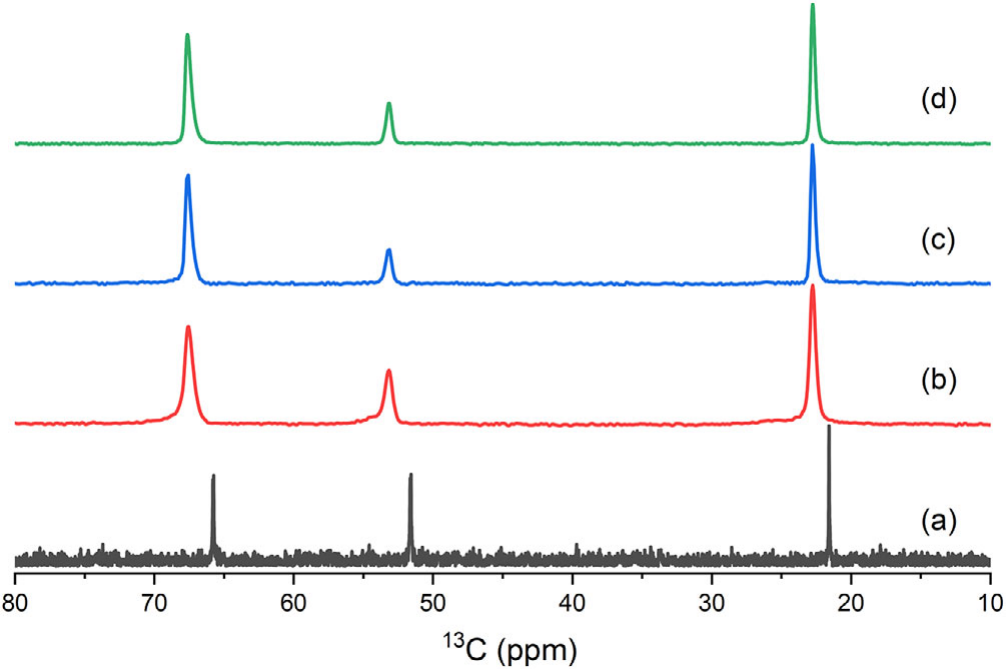
a) Solution‐phase ^13^C NMR spectrum of dimethylpyrrolidinium bromide dissolved in D_2_O, compared with b–d) ^13^C solid‐state CP MAS NMR spectra of ERS‐7, crystallized from gels containing (b) 1,6‐dibromohexane and tmdab, (c) polymer and dmpyrr and (d) only dmpyrr.

To prove that the cationic polymer is not required in all cases to crystallize ERS‐7, attempts were made to prepare ERS‐7 with a pre‐prepared dmpyrr OSDA in the absence of polymer **1,** using gels seeded with calcined ERS‐7. After some modification of gel chemistry (Table 1), it was possible to prepare highly crystalline ERS‐7 (Figures 9 and 10), which shows that the only OSDA required to template ERS‐7 in these preparations is the dmpyrr. Figure 11 compares the solid‐state ^13^C CP MAS NMR spectra of pure samples of as‐prepared ERS‐7 from the different synthetic routes with the solution‐phase NMR spectrum of as‐prepared dmpyrr. The ERS‐7 solid‐state ^13^C CP MAS NMR spectra are all very similar, suggesting they all contain the same OSDA, and the match with the solution‐phase NMR of dmpyrr indicates that in all cases this is the template. The small change in chemical shift between the dmpyrr in solution and the molecule encapsulated in the zeolite is commonly observed in the ^13^C NMR spectra of OSDAs, owing to the change in the long‐range environment. ^1^H NMR of the organic liberated upon dissolving the zeolite in aqueous HF confirms the assignment (Figure S1e).

**Figure 12:**
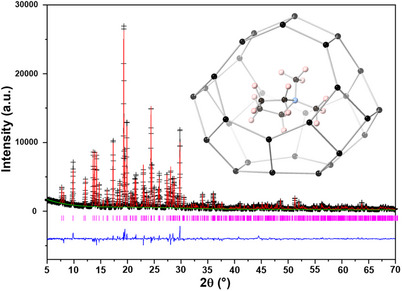
Rietveld plot of as‐prepared ERS‐7 (Cu K_α1_, 1.54056 Å) synthesized using tmdab and (inset) associated position of dimethylpyrrolidinium within esv cavities determined by refinement (note only 1 of 2 disordered, symmetry‐equivalent OSDA molecular sites is shown for clarity).

Furthermore, Rietveld refinement of PXRD data of the ERS‐7 prepared with tmdab /1,6‐bromohexane was performed. It was assumed that the OSDA was dmpyrr and this was allowed to refine as a semi‐rigid body. Figure 12 gives the Rietveld plot and crystal structure of the as‐prepared ERS‐7. The refinement gives an excellent fit and locates the dmpyrr in the 17‐hedral cavity with high occupancy (Table S4) while Na^+^ cations were located in 6‐membered rings shared by 11‐hedral cages. The *Pnma* space group results in two symmetry equivalent positions for the OSDA in the *esv* cavity, only one of which can be occupied per cage at any time, as shown.

Knowing that the OSDA is dmpyrr, the unit cell of the high silica ERS‐7 prepared with added tmdab is calculated to contain four OSDAs per unit cell and so with full occupancy of cages (combining elemental analysis and TGA data). Including EDS analysis this would give a unit cell composition as [C_4_H_8_N(CH_3_)_2_]_4_Na_1.1_Al_3.3_Si_44.7_O_96_.xH_2_O, although charge balance would require the presence of some Si vacancies with associated negative charge.

**Scheme 2:**
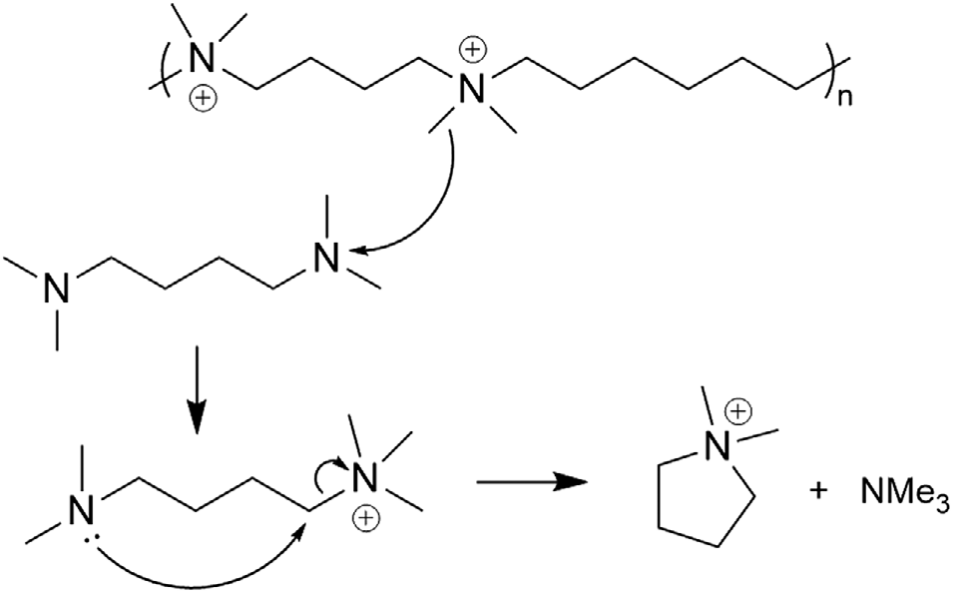
The suggested mechanism of formation of N,N‐dimethylpyrrolidinium by methylation and cyclization.

For the synthesis that starts with tmdab and gives the high silica ERS‐7, we, therefore, suggest that the diamine reacts to give the dmpyrr cation via intramolecular nucleophilic substitution under the highly alkaline reaction conditions. Reactivity of amines added to zeolite synthesis gels has been reported previously^[^ ^42^ ^]^ and catalyzed cyclization of α,ω‐diaminobutane to pyrrolidine has been observed.^[^ ^38^ ^]^ We hypothesize that the cyclization mechanism involves initial methylation of one end of the diamine via equilibration with the methylated polymer, to give a suitable trimethylamine leaving group for the nucleophilic substitution (Scheme 2).

The addition of cationic polymer **1** in addition to dmpyrr or its precursors has a strong effect in these syntheses, enabling pure ERS‐7 to be prepared in the absence of seed crystals, even though the polymer is not included in the final product. In the unseeded preparations, its absence results in the crystallization of mordenite, which is a favored product of the sodium aluminosilicate gel. The presence of polymer therefore allows the added OSDA to direct the crystallization of the gel to ERS‐7 crystals by inhibiting the mordenite crystallization. Notably, SEM indicates an increase in the crystal size of ERS‐7 as the amount of polymer is reduced in the presence of dmpyrr OSDA (Figure 10). This suggests that the polymer increases the nucleation rate of ERS‐7 relative to its growth rate while inhibiting the nucleation of mordenite. To understand better the selective inhibition effect of the polymer on the mordenite compared to the ERS‐7, the configuration of an oligomeric unit was modeled within the competing zeolite phases. In each case, the inclusion of the oligomer was found to be energetically feasible, even though it was observed that this had not happened in the final product. Within the **ESV** framework, the hypothetical low‐energy configuration ran between cavities, through 8R windows, and with a zig‐zag arrangement (Figure S13), as reported previously in modeling studies of this oligomer in the **ITH** framework.^[^ ^19^ ^]^ In the **MOR** framework, this zig‐zag arrangement was not possible, and instead, the oligomer adopted a different form along the large 12R channels. We therefore speculate that if this zig‐zag arrangement is a favorable one under these conditions, it might act to nucleate ERS‐7 but not mordenite.

Other authors have considered the role of a cationic polymer in zeolite synthesis. Zhou et al. draw upon crystallization kinetics and electron microscopic studies of the zeolite morphology to suggest that the polymer inclusion results in a prolonged pre‐nucleation induction period as inorganic precursors arrange themselves around the polymer, followed by nucleation and then growth of semicrystalline nuclei.^[^ ^17^ ^]^ It may be that the polymer in these syntheses of ESV plays a similarly important role in the pre‐nucleation induction period and the subsequent nucleation.

## 3 Conclusion

*N*,*N*‐dimethylpyrrolidinium is an effective OSDA for the synthesis of high silica ERS‐7 (Si/Al = 10.5–14), which has previously only been prepared in fluoride‐containing syntheses. The zeolite is stable to calcination and ion exchange and shows CO_2_ adsorption properties similar to those reported previously for high silica ERS‐7.

The new OSDA can be pre‐prepared and added during gel preparation, but we have also shown that it can be generated in situ by the cyclization of *N*,*N*,*N’*,*N’*‐tetramethyl‐1,4‐diaminobutane. The cyclization might occur via methylation of the tmdab from the cationic polymer followed by intramolecular nucleophilic substitution and elimination of trimethylamine. Although there are a few literature suggestions that diamines react during zeolite synthesis and that the products can act as OSDAs for zeolite crystallization, the example described here is strongly supported by experimental evidence. The organic conversion and templating are highly specific and provide a convenient route to a cyclic alkylammonium product that is an effective template. It can inspire future one‐pot in‐situ OSDA syntheses and zeolite crystallizations.

The cationic polymer, poly‐dimethylammoniumbutane, dimethylammoniumhexane promotes the crystallization of ERS‐7 rather than mordenite in unseeded preparations in this system, which otherwise give mordenite. We hypothesize that in addition to methylation of the diaminobutane to give a good leaving group during cyclization, it disfavors the nucleation of mordenite compared to ERS‐7 when it interacts with inorganic precursors in the gel prior to crystal growth. This can be of practical use in investigating new organocations as OSDAs for the discovery of novel zeolites.

## 4 Experimental Section

Zeolite ERS‐7 was initially synthesized from the gel composition: 22 Na_2_O: 1.0 Al_2_O_3_: 60 SiO_2_: 14 C_8_H_20_N_2_: 12 C_6_H_12_Br_2_: 2670 H_2_O, in which the aim was to prepare the polymer in situ by the reaction of 1,6‐dibromohexane and *N*,*N*,*N’*,*N’*‐tetramethyl‐1,4‐diaminobutane (tmdab). Additionally, ERS‐7 could be prepared in the presence of pre‐made polymer using the gel composition: 22 Na_2_O: Al_2_O_3_: 60 SiO_2_: 2 C_8_H_20_N_2_: 12 (‐N(CH_3_)_2_(CH_2_)_4_N(CH_3_)_2_(CH_2_)_6_Br_2_‐): 2670 H_2_O. The synthesis gel was prepared by dissolving NaOH (11 mmol) and Al(NO_3_)_3_.9H_2_O (0.5 mmol) in deionized water (15–25 mL). Ludox AS‐40 (30 mmol of SiO_2_) was added and the mixture was stirred for 2 hours. After tmdab (1.0–7.0 mmol) and 1,6‐dibromohexane (0.0–6.0 mmol) or cationic oligomer (6 mmol of repeat unit) were added, the mixture was stirred for a further 3 h. The gel was aged at RT for 24 h under static conditions. The crystallization was carried out in a Teflon‐lined stainless‐steel autoclave at 433 K for 7 days with tumbling at 60 rpm. The solid product was recovered by filtration, washed repeatedly with deionized water, and then dried overnight at room temperature.

Additional syntheses were performed using the same reaction variables of temperature and time, with some changes in the silica source and gel composition. The organic additive was replaced with one of the following:N,N,N'N'‐tetramethyl‐1,6‐diaminohexane or *N*,*N*‐dimethylpyrrolidinium (dmpyrr) bromide (synthesis in SI), all with or without the addition of the cationic polymer 1. Details on exact conditions and the PXRD patterns of the ERS‐7 products are given in the Supporting Information.

The as‐prepared ERS‐7 with in situ cationic polymer formation was calcined at 823 K in oxygen for 12 h to remove all organic molecules. The resulting Na,H‐ERS‐7 was fully exchanged to the ammonium form with 3 m ammonium chloride solution at 333 K, eight times for 5 h, until no sodium could be observed by EDX analysis. Subsequently, the ammonium form was converted to the sodium form by repeated extended cation exchange treatments at 353 K using 10 wt.% sodium nitrate solution.

The crystallinity of all samples was confirmed by laboratory PXRD using a PANalytical Empyrean diffractometer with a Cu X‐ray tube (Cu K*_α_*_1_, 1.54056 Å) and X'celerator RTMS detector. The diffractometer was operated in Bragg‐Brentano reflection geometry, θ‐2θ mode, at room temperature. To determine the structure of dehydrated zeolites, the powders were loaded into 0.7 mm quartz capillaries and dehydrated at 623 K at 5×10^−5^ mbar on a glass vacuum line for 10 h. For the dehydrated samples, a STOE STADIP diffractometer with Mo K_α1_ X‐radiation (0.70926 Å) operated in capillary Debye‐Scherrer mode was used. Scanning electron microscopy (SEM) imaging and energy dispersive X‐ray spectroscopy (EDS) were performed on Jeol JSM IT800 and Jeol JSM IT200 scanning electron microscopes, equipped with a Jeol DrySD detector.

Thermogravimetric analysis (TGA) of the samples was performed using a Stanton Redcroft STA‐780, a NETZSCH TG1000 M, or a NETZSCH STA 449 with a heating rate of 5 K min^−1^ up to 1073 K in flowing air.

Solution‐phase NMR spectra were collected on either a Bruker AVIII 500 or a Bruker AVII 400 spectrometer. Solid‐state magic angle spinning (MAS) NMR spectra were recorded using a Bruker Avance III spectrometer equipped with a wide‐bore magnet operating at magnetic field strength, B_0_, of 9.4 T (Larmor frequencies of 400.1 MHz, 100.6 MHz, 40.6 MHz, 104.3 MHz, and 79.5 MHz for ^1^H, ^13^C, ^15^N ^27^Al and ^29^Si, respectively). Samples were packed into standard ZrO_2_ rotors with outer diameters of 4 mm and rotated about an axis inclined at the magic angle at a rate of 5 kHz (^15^N) 10 kHz (^29^Si), 12.5 kHz (^13^C) or 14 kHz (^27^Al) using Bruker MAS probes. Chemical shifts are quoted in ppm relative to (CH_3_)_4_Si (^13^C and ^29^Si), CH_3_NO_2_ (^15^N), and 1 M Al(NO_3_)_3_ (aq) using secondary solid references of forsterite (β‐Mg_2_SiO_4_, δ = −62 ppm), l‐alanine (δ(CH_3_) = 20.5 ppm), ^15^N‐enriched glycine (δ = −347.4 ppm) and Al(acac)_3_ (δ_iso_ = 0.0 ppm). The ^13^C and ^15^N NMR spectra of ERS‐7 were recorded using cross polarisation (CP) from ^1^H with a contact pulse (ramped for ^1^H) of 0.5 ms (^13^C) and 5 ms (^15^N). Signal averaging was carried out for 1024 (^13^C) and 51200 (^15^N) transients with a recycle interval of 3 s. High‐power (ν_1 _≈ 100 kHz) TPPM‐15 ^1^H decoupling was applied during acquisition in both cases. The ^27^Al spectra were recorded with signal averaging for 2048 transients for as‐made ERS‐7 and 12288 transients for H‐ERS‐7 and with a recycle interval of 1 s. A short flip angle (β = 17^о^) was used to enable quantitation. The ^29^Si MAS NMR spectra were recorded with signal averaging for 552 transients for as‐made ERS‐7 and 496 transients for H‐ERS‐7 with a recycle interval of 120 s.

The crystal structures were determined by Rietveld refinement against the PXRD data using TOPAS Academic software.^[^ ^34^ ^]^ The starting framework model for ERS‐7 (ESV) was adapted from the literature with the unit cell modified to that derived from the diffraction patterns. The fractional occupancies and atomic coordinates for the extra framework cations were refined. The framework atomic positions were initially refined with geometric restraints on T‐O (T sites treated as Si; 1.61 Å) and O‐O (2.61 Å) distances to maintain regular tetrahedral coordination. To improve T‐O‐T angle geometry where required, an additional T‐T restraint was added (3.14 Å). A further penalty was introduced for Na^+^ cations approaching framework O sites for Na‐O distances < 2.5 Å. OSDA species were treated as rigid bodies, and the orientation and position within cavities were refined as a single structure. Atomic scattering factors were used for all atoms. The peaks were fitted with a Pseudo‐Voigt profile and simple axial asymmetry model and backgrounds were fitted by up to 9‐term shifted Chebyshev functions. The crystallographic data for the ERS‐7 structures are given in the Supporting Information and cif files which have also been deposited at the Cambridge Crystallographic Data Centre.^[^ ^43^ ^]^

High‐pressure CO_2_ adsorption isotherms from 0–10 bar at 298 K were measured gravimetrically on a Hiden Intelligent Gravimetric Analyzer (IGA). All samples were activated at 553 K for 10 h prior to measurements. The mass change for each adsorption/desorption step was monitored, and a final reading was taken when it had reached 98% of the asymptotic equilibrium value or after 90 min, whichever was shorter.

Molecular modeling of cationic polymer **1**, as well as the “linear” and “cyclic” tmdab was initially performed in Materials Studio by BIOVIA using either the Forcite or Adsorption Anneal modules.^[^ ^36^ ^]^ COMPASS III was the forcefield used for all calculations. The charges on the framework atoms and OSDAs were forcefield‐assigned initially. An opposing charge was spread across all the framework atoms to balance the positive charge of the OSDA cations docked into the structure. The empty silica framework structure of ESV was taken from the IZA Database.^[^ ^26^ ^]^ It was converted to *P*1 symmetry before any other modifications or calculations. The optimal positions and orientations of OSDAs in the ERS‐7 unit cell were determined through a “Simulated annealing” calculation, where a Monte Carlo (MC) type approach is employed when they were not positioned manually inside the structure. The MC calculation was run with 100000 loading steps, 10 heating cycles, and 500,000 steps per cycle. The start and final temperatures were set to 300 K and 700 K, respectively. The output structures were geometry optimized. The Ewald summation method was used for the calculation of electrostatic terms. The atom‐based approach was used for the van der Waals terms, with a cut‐off distance of 15.5 Å. The Smart algorithm was used for the geometry optimization and the convergence criteria were Δenergy < 10^−4^ kcal mol^−1^, Δforce <5 × 10^−3^ kcal mol^−1^ Å^−1^, stress <5 × 10^−3^ GPa, displacement <5 × 10^−5^ Å. The lowest energy structure was also subjected to further geometry optimization and dynamics calculations. The unit cell and atomic positions were allowed to be optimized during the geometry optimization calculations. The dynamics calculations were carried out in the NVE ensemble, at 650 K. Initial velocities were randomly assigned. The simulations were run with a 1 fs timestep, for 50 ps, with structures generated every 500 steps. Consequently, 101 structures were generated as input structures for subsequent geometry optimization calculations. Only structures that converged within 5000 steps were considered for further calculations or as final results.

A comparison of the stabilization energy conferred by 4 molecules of dmpyrr and dmpip in ERS‐7 was performed by using Orb V2.^[^ ^40^, ^41^ ^]^ This Machine‐Learned Force Field (MLFF) allowed calculations at the PBE‐D3 level at a fraction of the computational cost. The initial ESV structures for this modeling were determined with the geometry optimization and dynamics calculations described above. Then, the location of the 4 Al atoms for balancing the charge on the OSDAs was determined with an MC approach (in which non‐Löwenstein positions were disfavoured). The geometry‐optimized OSDAs were placed in the structure through an algorithm that minimized overlap with the framework. All geometry optimizations were carried out with the BFGS algorithm with F_max_ < 0.01 eV Å^−1^ conversion criterion. All calculations were executed in Python using the Atomic Simulations Environment (ASE) and RDKit.^[^ ^44^, ^45^ ^]^

## Conflict of Interests

The authors declare no conflict of interest.

## Supporting information



Supporting Information

## Data Availability

The data that support the findings of this study are openly available in PURE repository at St Andrews University at https://doi.org/10.17630/46025e62‐37ec‐4907‐8e67‐1f77543eab7d, reference number 313407318.
