# Supplementary material for: In Situ Generation by Cyclization of an Organic Structure Directing Agent for the Synthesis of High Silica Zeolite ERS‐7
Source: Chemistry. 2025 May 7;31(30):e202500327. doi: 10.1002/chem.202500327 (PMC12117171; doi:10.1002/chem.202500327)
Supplement: Supplementary file 1 — Supporting Information [file CHEM-31-e202500327-s001.docx]

**Supporting Information**

**In Situ Generation by Cyclisation of the Organic Structure Directing Agent for the Synthesis of a High Silica ESV Zeolite**

Magdalena M. Lozinska, Ruxandra G. Chitac, Elliott L. Bruce, Malavika Manoj, Yuanyuan Du, Daniel M. Dawson, Sharon E. Ashbrook, Paul A. Cox and Paul A. Wright

### S1. Organic synthesis

S1.1 Polymer synthesis

S1.2 Synthesis of N,N-dimethylpyrrolidinium bromide

S2. Zeolite synthesis and general characterisation

S2.1 Zeolite synthesis and PXRD characterisation

S2.2 N_2_ adsorption of calcined ERS-7

S2.3 ^15^N MASNMR of as-prepared ERS-7

S2.4 TGA of as-prepared ERS-7

S3. Modelling tmdab in pure silica ESV

S4. Low polymer/SiO_2_ : dmpyrr synthesis of mordenite

S5. Crystallography of ERS-7 prepared using tmdab and 1,4-dibromobutane

S6. Modelling of dmpip and dmpyrr in ERS-7

S7. Oligomers of cationic polymer **1** energy minimised within ESV and MOR frameworks

S8. References

### **S1. Organic synthesis**

### **S1.1 Polymer synthesis**

The cationic polymer, **1** (see Figure 1a), was prepared by dissolving *N,N,N′,N′*-tetramethyl-1,4-diaminobutane (tmdab, 30 mmol) and 1,6-dibromohexane (30 mmol) in 50 mL of ethanol and refluxing for 6 h. The white precipitate was filtered, washed with diethylether and dried at RT overnight. The solution-phase ^13^C NMR and ^1^H NMR spectra of the cationic polymer are given below (Figures 1a and 1b). All peaks are assigned, indicating no significant impurity.

**^13^C NMR:** $\delta_{C}$ (126 MHz, D_2_O) 19.2(C1), 21.94 (C2), 25.22 (C3), 50.32 (C1), 63.29, 64.40 (C5,C6).

**^1^H NMR:** $\delta_{H}$ (500 MHz, D_2_O) 1.45 (4 H, m, H3), 1.82,1.87 (4 H, m, 4H, m H2, H3), 3.09 (12 H, s, H4), 3.34, 3.40 (4 H, m, 4H, m, H5, H6).

.
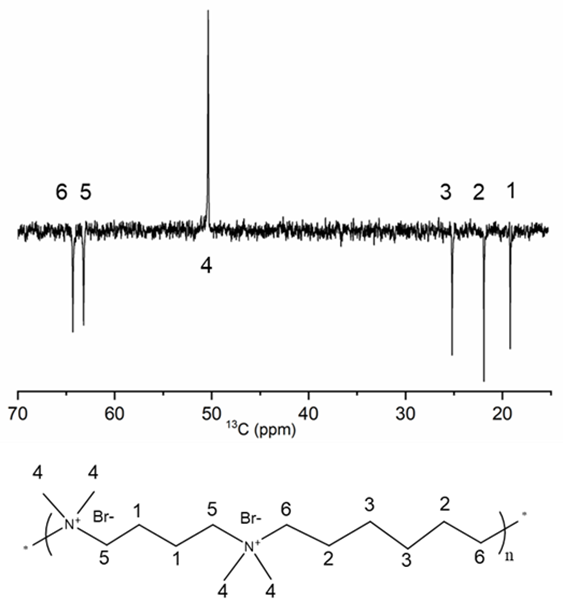


**Figure S1a.** Solution-phase ^13^C NMR spectrum of cationic polymer **1**, with numbering scheme and assignment shown.


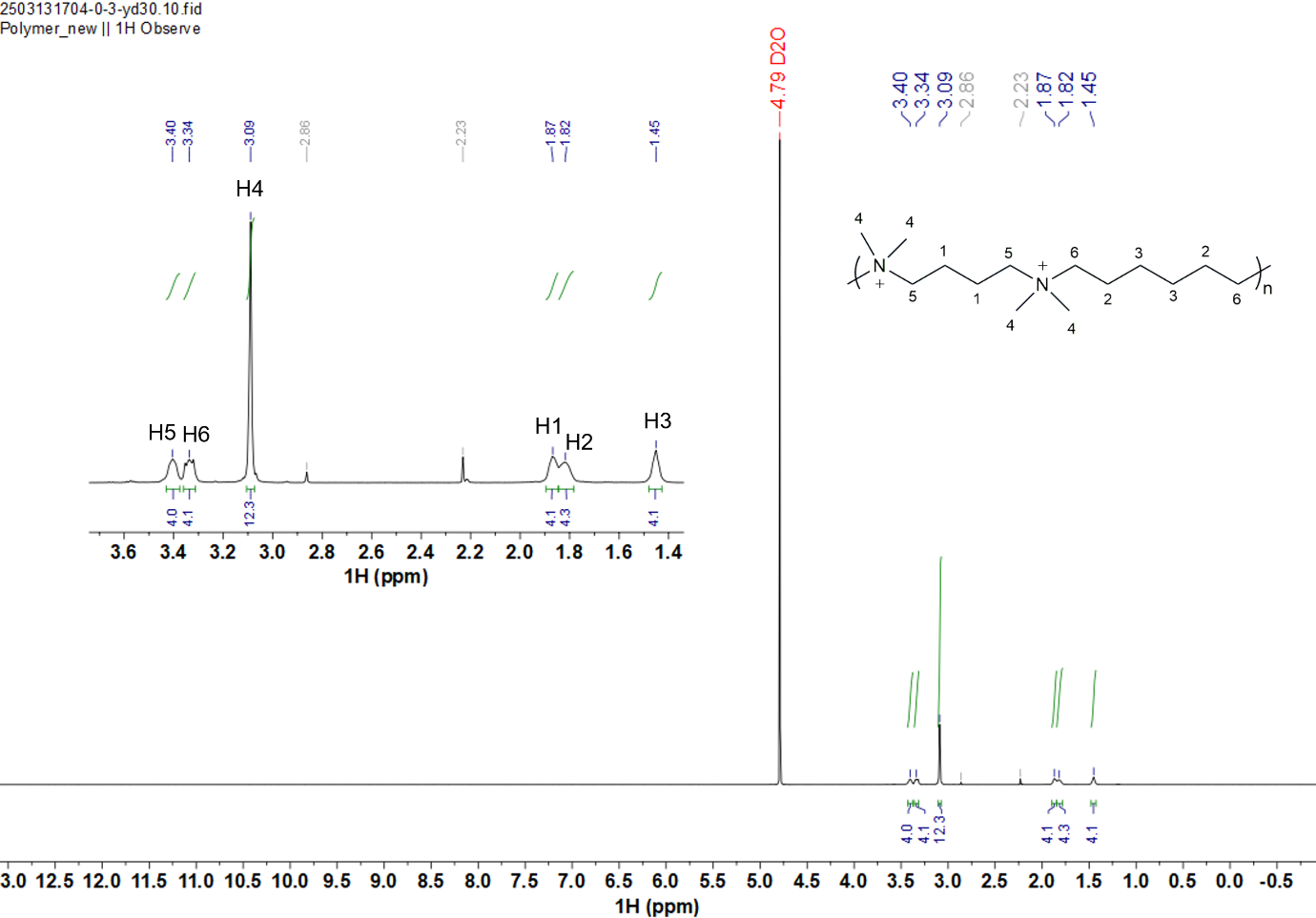


**Figure S1b.** Solution-phase ^1^H NMR spectrum of cationic polymer **1**, with numbering scheme and assignment shown.

**S1.2 Synthesis of *N,N*-dimethylpyrrolidinium bromide**





21.1 g (51.4 mmol) dimethylamine (ca. 11% in ethanol) was dissolved in 12.3 ml of ethanol under ice bath conditions, followed by the addition of 8.5 g (61.7 mmol) anhydrous potassium carbonate. 11.1 g (51.4 mmol) 1,4-dibromobutane was added dropwise to the mixture. The reaction mixture was returned to room temperature once all reagents had been added. The mixture was then heated under reflux for 36 hours. The potassium carbonate was separated by filtration. The solvent from the filtrate was removed using a rotary evaporator, and the resulting solid was washed with cold acetone and diethyl ether. The white powder product (7.8 g, 60% yield) was dried at 75 °C overnight and then analysed by NMR spectroscopy (Figures 1c and 1d). All peaks are assigned, indicating no significant impurity.

**^1^H NMR:** $\delta_{H}$ (500 MHz, D_2_O) 2.12 (4 H, m, H3), 3.03 (6 H, s, H1), 3.40 (4 H, t, H2).

**^13^C NMR:** $\delta_{C}$ (126 MHz, D_2_O) 21.5 (C3), 51.6 (C1), 65.7 (C2).


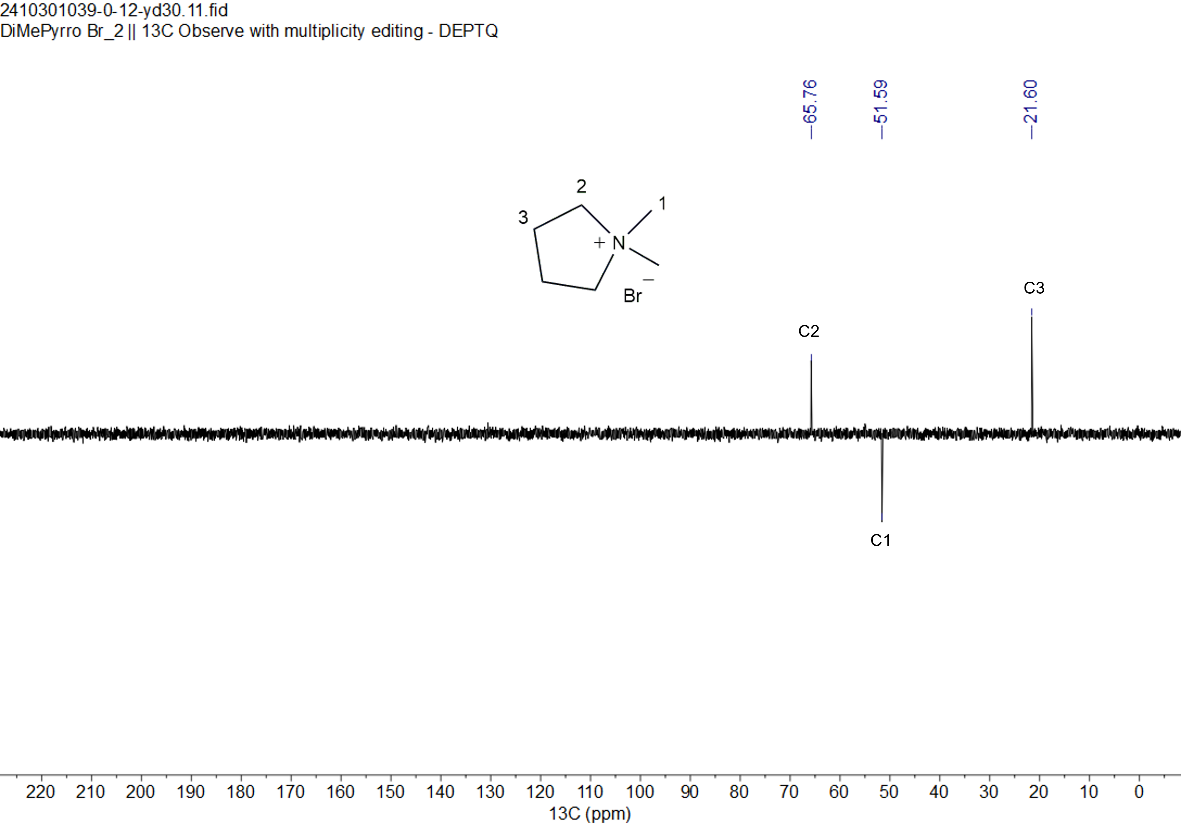


**Figure S1c.** Solution-phase ^13^C NMR spectrum of N,N-dimethylpyrrolidinium bromide, with numbering scheme and assignment shown.

**
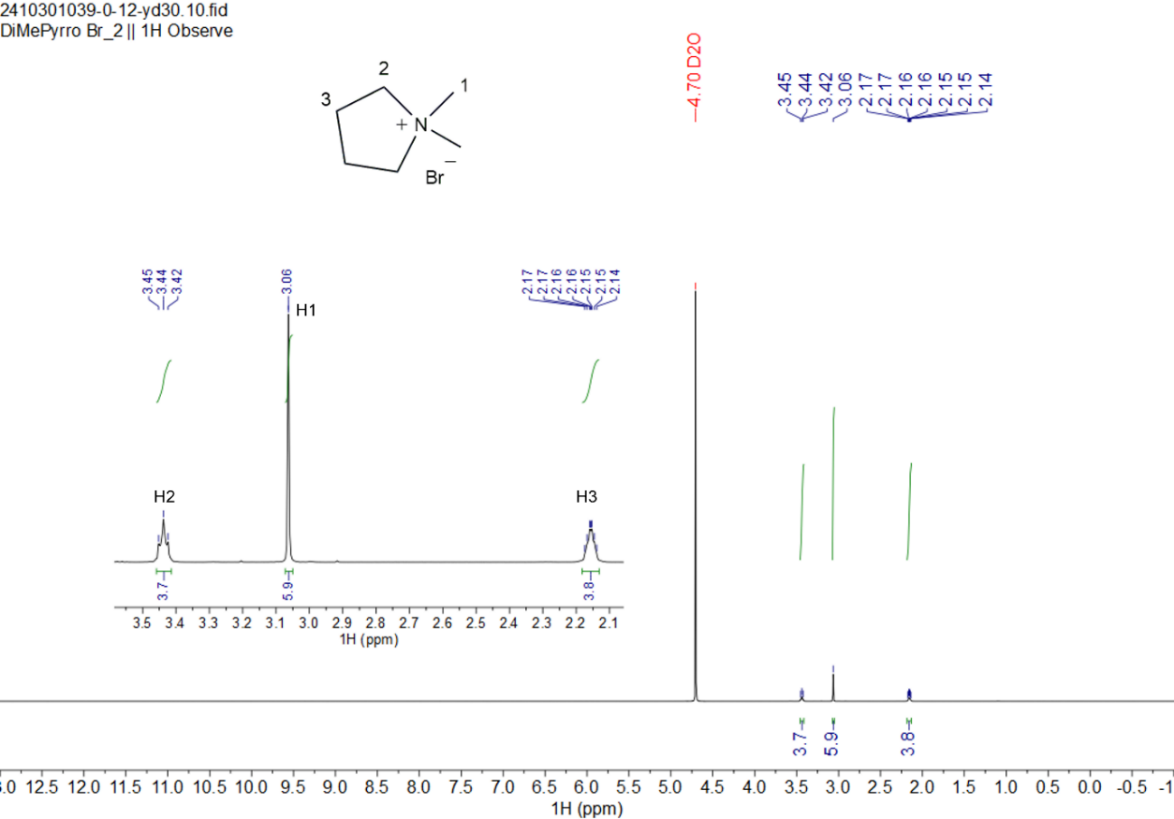
**

**Figure S1d.** Solution-phase ^1^H NMR spectrum of N,N-dimethylpyrrolidinium bromide, with numbering scheme and assignment shown.


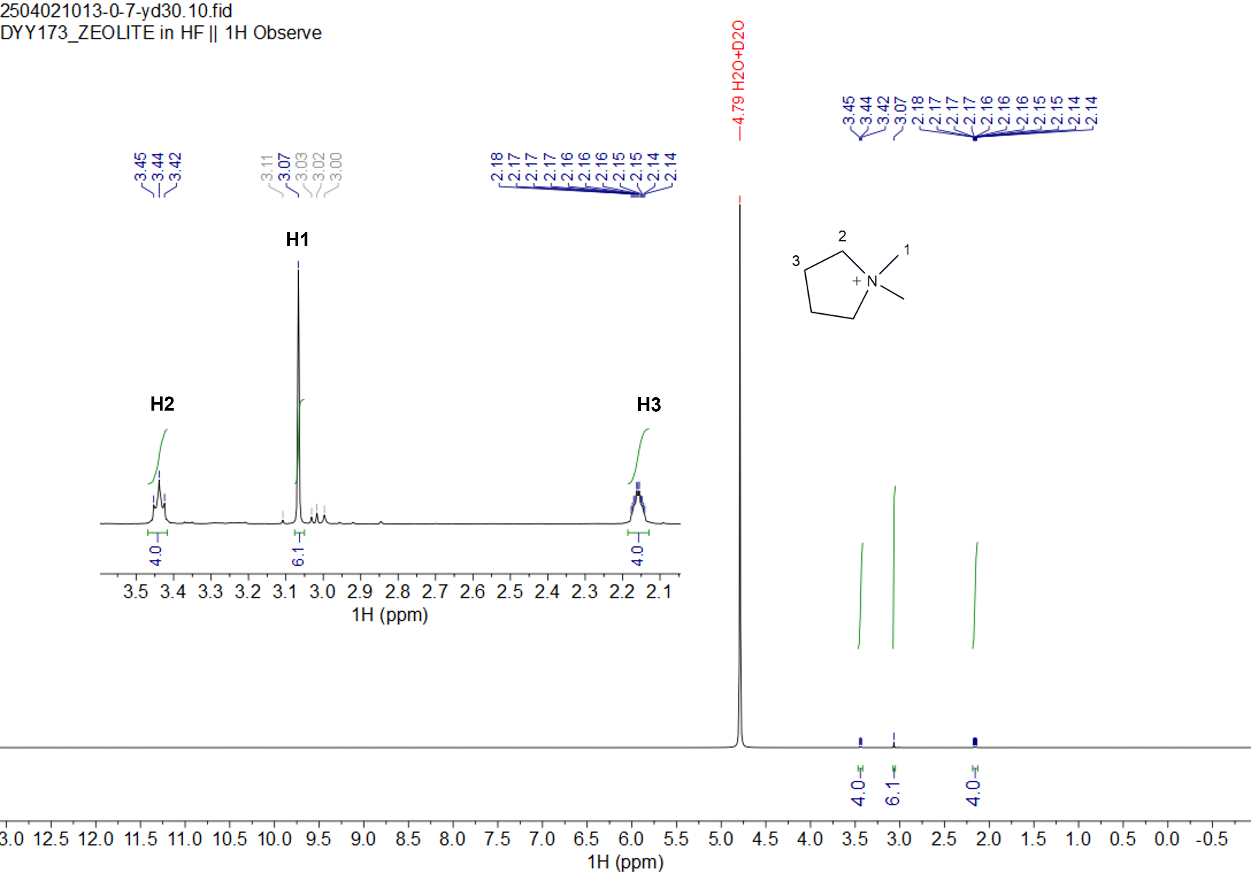


**Figure S1e.** ^1^H NMR spectrum of solution obtained from dissolving 50 mg as-synthesised ERS-7 zeolite in 0.5 ml HF(48 wt%) (Care with handling: HF is highly toxic) followed by neutralisation in 5M NaOH in D_2_O.

**S2. Zeolite synthesis and general characterisation**

**S2.1 Zeolite synthesis**

Zeolite ERS-7 was synthesised from these molar gel compositions: 22 Na_2_O: 1.0 Al_2_O_3_: 60 SiO_2_: 14 C_8_H_20_N_2_: 12 C_6_H_12_Br_2_: 2670 H_2_O or 22 Na_2_O: 1.0 Al_2_O_3_: 60 SiO_2_: 2 C_8_H_20_N_2_: 12 oligomer dibromide : 2670 H_2_O. The starting mixture was prepared by dissolving NaOH (44 mmol) and Al(NO_3_)_3_.9H_2_O (2 mmol) in deionized water (15-25 ml). Then Ludox AS-40 (60 mmol of SiO_2_) was added and the mixture was stirred for 2 hours. After tmdab (2.0-14.0 mmol) and 1,6-dibromohexane (0.0-12.0 mmol), or cationic oligomer (12 mmol), were added the mixture was stirred for further 3 hours. The gel was aged at RT for 24 hours. The crystallisation was carried out in a Teflon-lined stainless steel autoclave at 433 K for 7 days with agitation provided by rotation at 60 rpm.

**
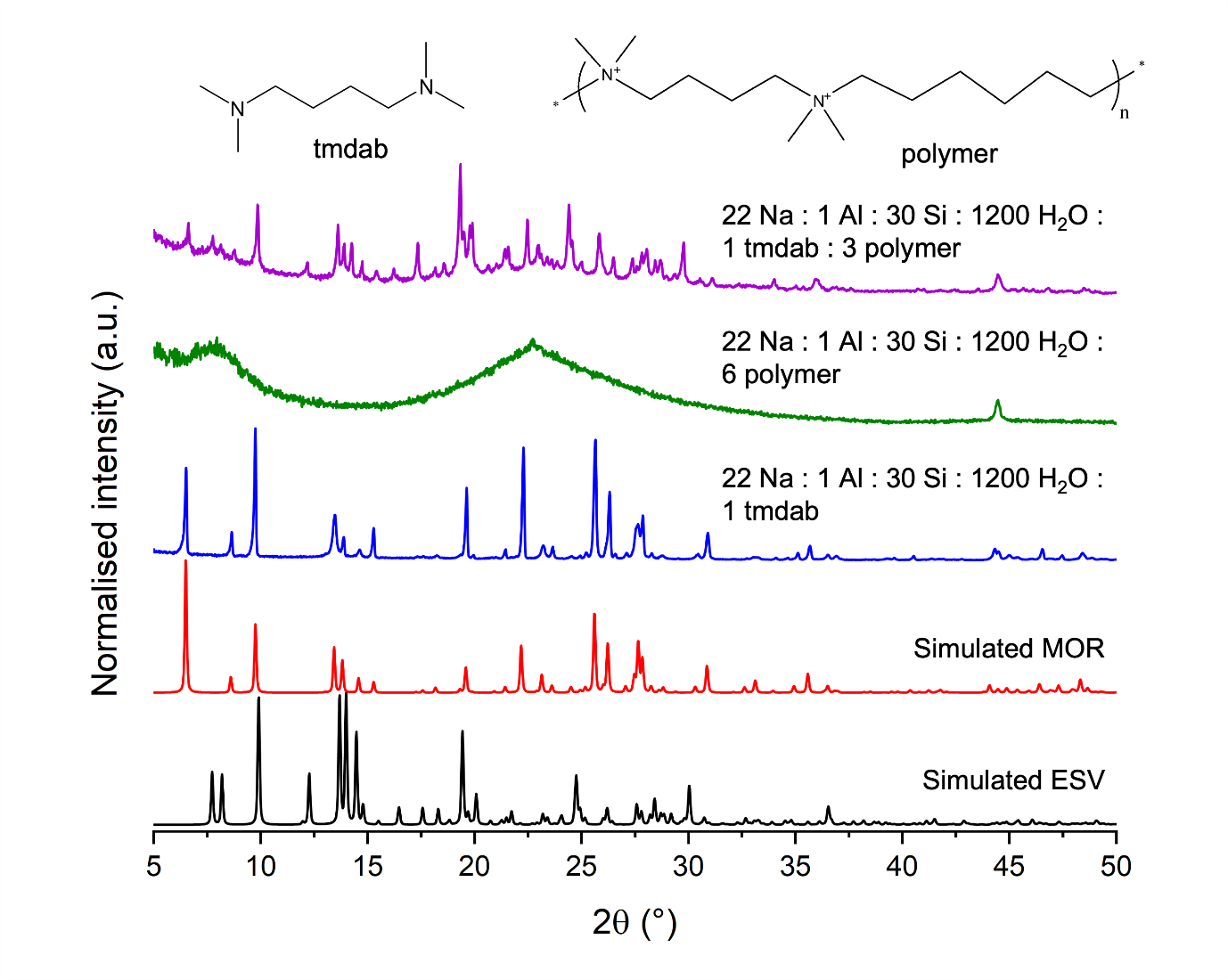
Figure S2.** PXRD patterns (Cu K_α1_, 1.54056 Å) of products of hydrothermal reactions additional to those of Table 1 in the main paper including, as organic additive, only *N,N,N′,N′-*tetramethyldiaminobutane (tmdab) (blue), only cationic polymer (green) or tmdab with a reduced amount of polymer compared to that which gives ERS-7 (purple) as documented in Table 1. Gel compositions given in molar ratios. Simulated patterns use data from the Atlas.[1]


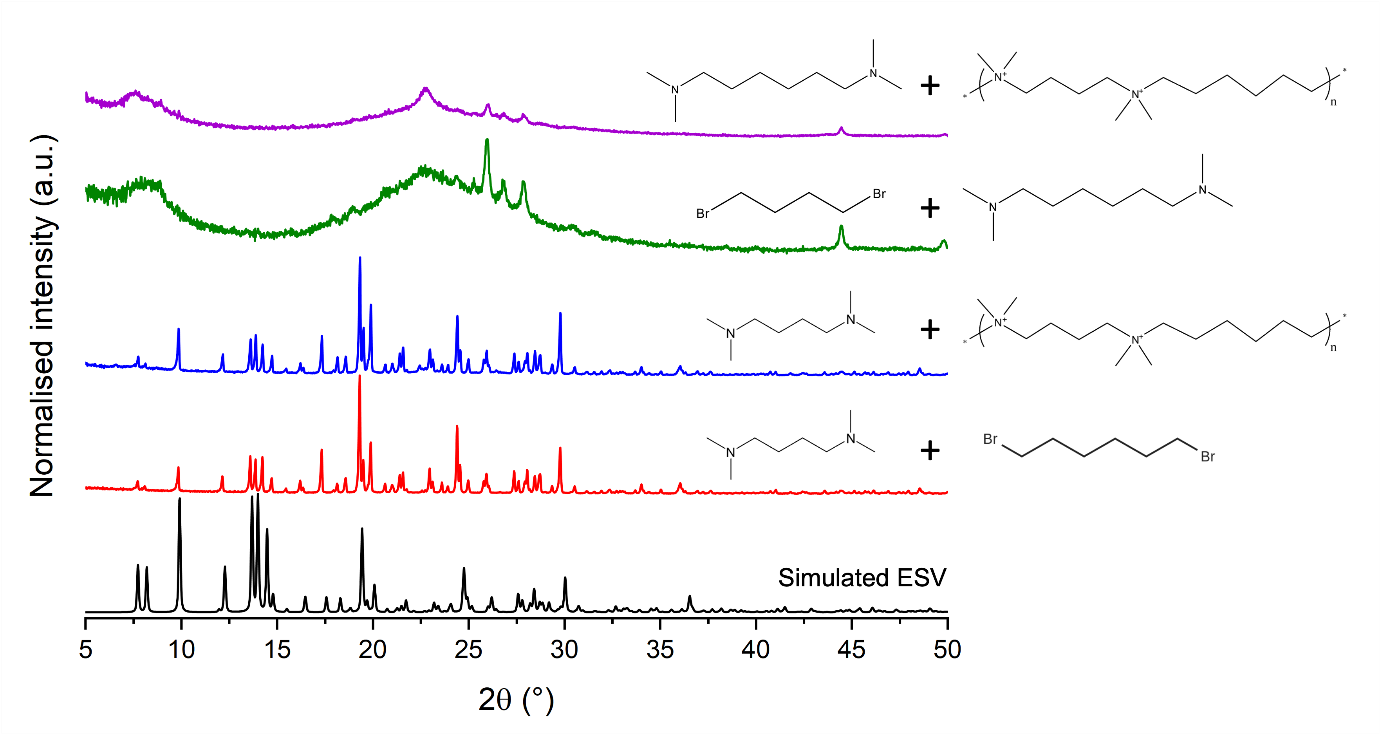


**Figure S3.** PXRD (Cu K_α1_, 1.54056 Å) of products of hydrothermal reactions including (c) *N,N,N′,N′-*tetramethyldiamino*hexane* (tmdah) plus 1,4-dibromobutane (6:7) or (d) tmdah plus cationic polymer **1**, compared to crystallisations under similar conditions (as in Table 1 of main paper) using tmdab with (a) a mixture of tmdab and diBrC6, (b) an equivalent amount of pre-made polymer, as described in Table S1 below. These data show that tmdab (rather than tmdah) is required for the crystallisation of ERS-7.

**Table S1.** Gel compositions for products shown in Figure S3.

| Organic additives | Na : Al : Si : H_2_O | dibromo-alkane | Tetramethyl-diamine | Polymer  (repeat) |
| --- | --- | --- | --- | --- |
| (a) tmdab + diBrC6 polymer precursors | 22 : 1 : 30 : 1200 | 6 dibromohexane | 6 tmda-butane | 0 |
| (b) polymer + tmdab | 22 : 1 : 30 : 1200 | 0 | 1 tmda-butane | 6 |
| (c) tmdab + diBrC6 polymer precursors | 22 : 1 : 30 : 1200 | 6 dibromobutane | 6 tmda-hexane | 0 |
| (d) polymer + tmdah | 22 : 1 : 30 : 1300 | 0 | 1 tmda-hexane | 6 |

**
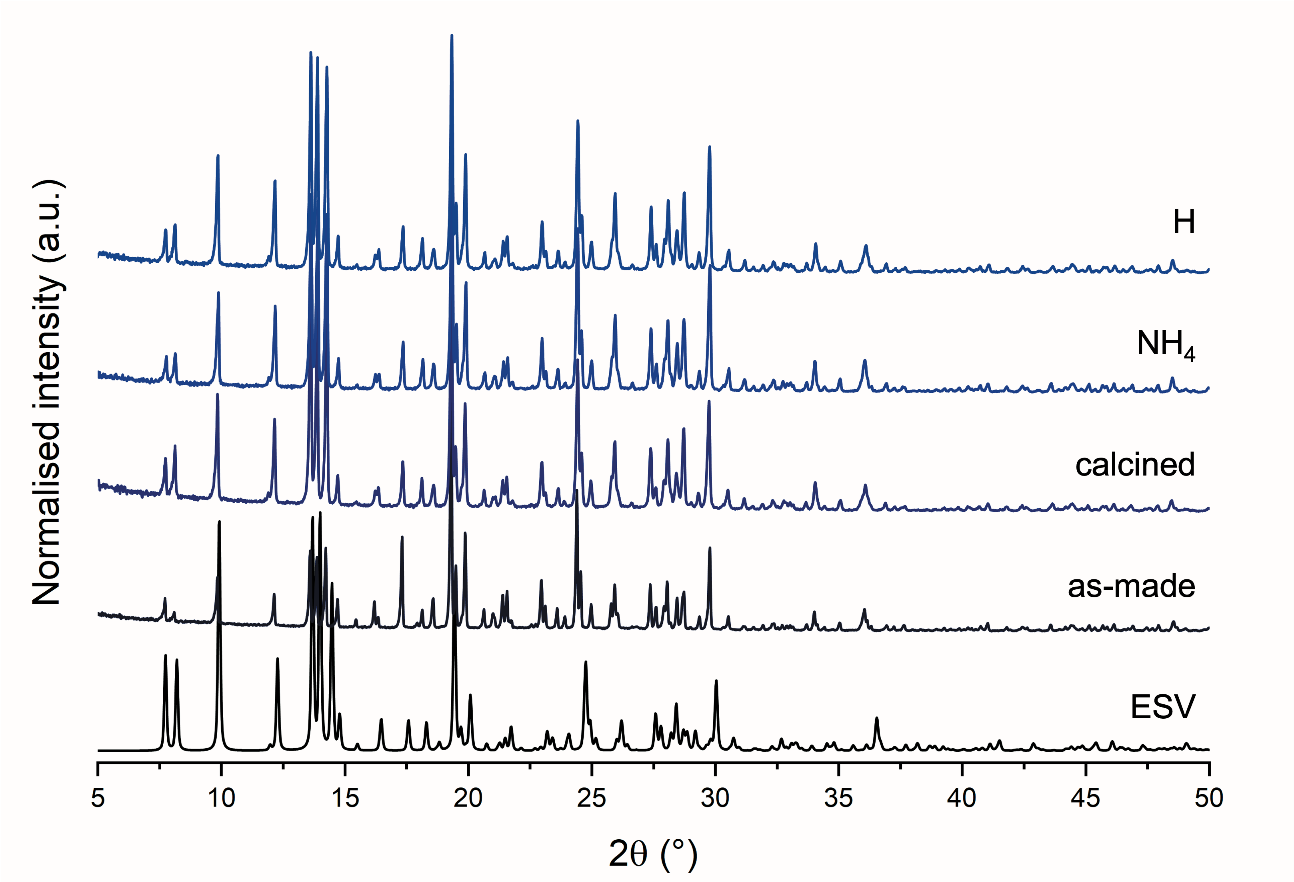
**

**Figure S4.** PXRD patterns: simulated pattern of pure silica ERS-7 (ESV),[1] compared to ERS-7 (prepared in this work using tmdab and cationic polymer as additives according to Table 1) in as-made, calcined, NH_4_- and H-forms

**S2.2 N_2_ adsorption at 77 K on calcined ERS-7**


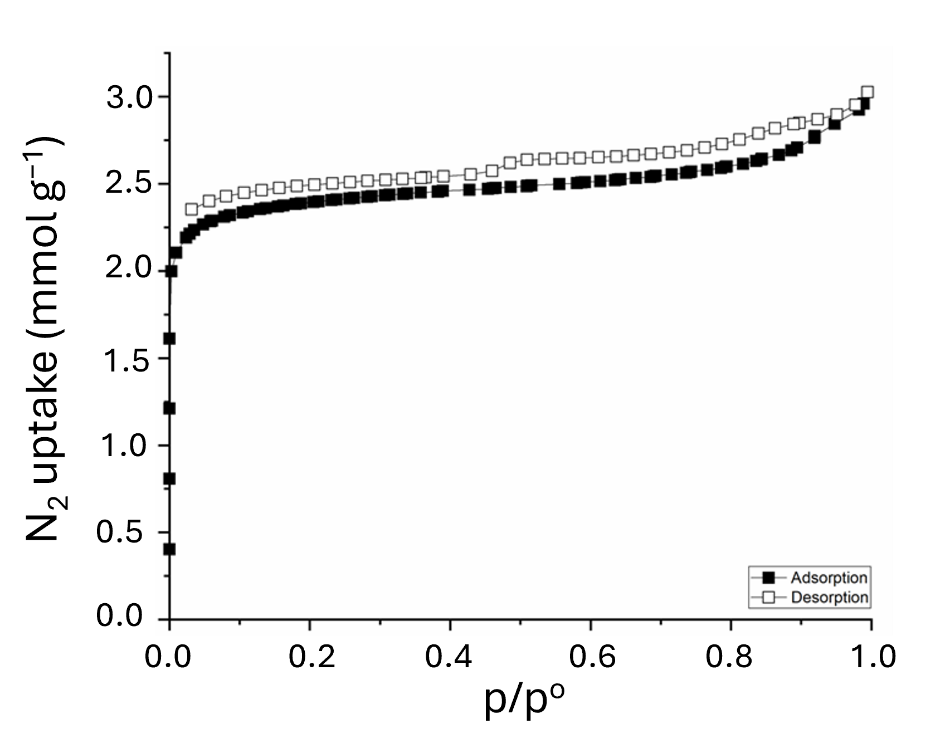


**Figure S5.** N_2_ adsorption at 77 K of calcined ERS-7 prepared with tmdab and dibromohexane added to the synthesis gel.

**S2.3 ^15^N MASNMR of as-prepared ERS-7**


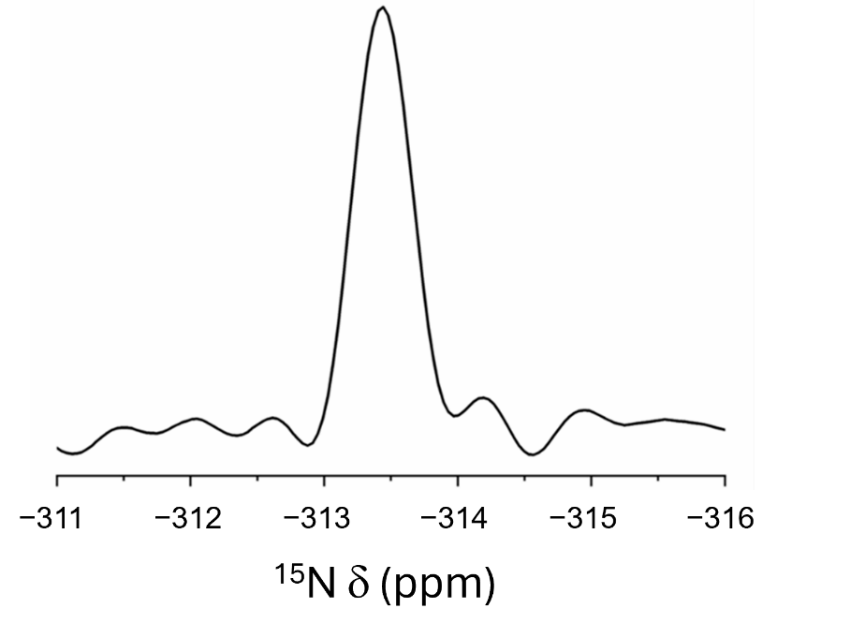


**Figure S6.** ^15^N CP MAS NMR spectrum of ERS-7 prepared with tmdab and dibromohexane added to the synthesis gel.

**S2.4 TGA of as-prepared ERS-7**


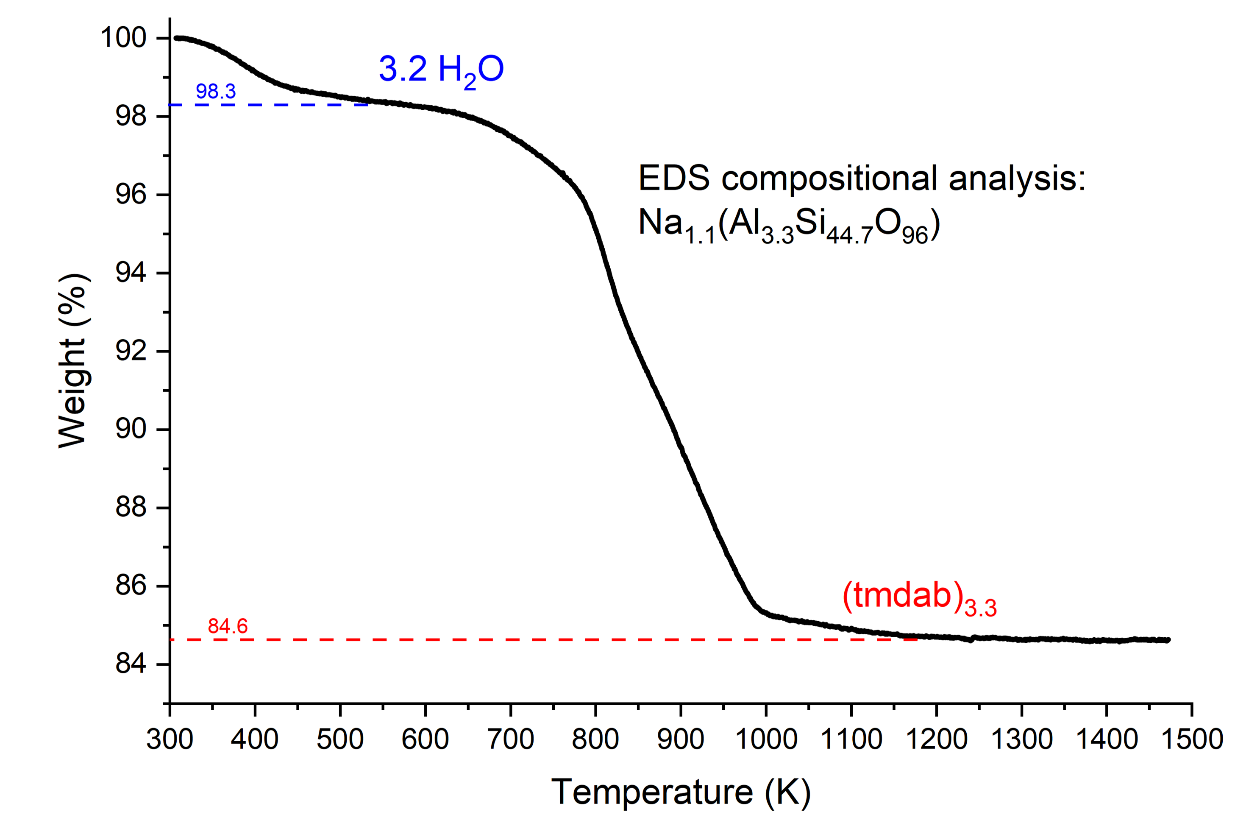


**Figure S7.** TGA weight loss curve of ERS-7 prepared with tmdab and dibromohexane added to the synthesis gel, labelled with the plateau values considered for the loss of H_2_O and OSDA.

**S3. Modelling tmdab in pure silica ESV**


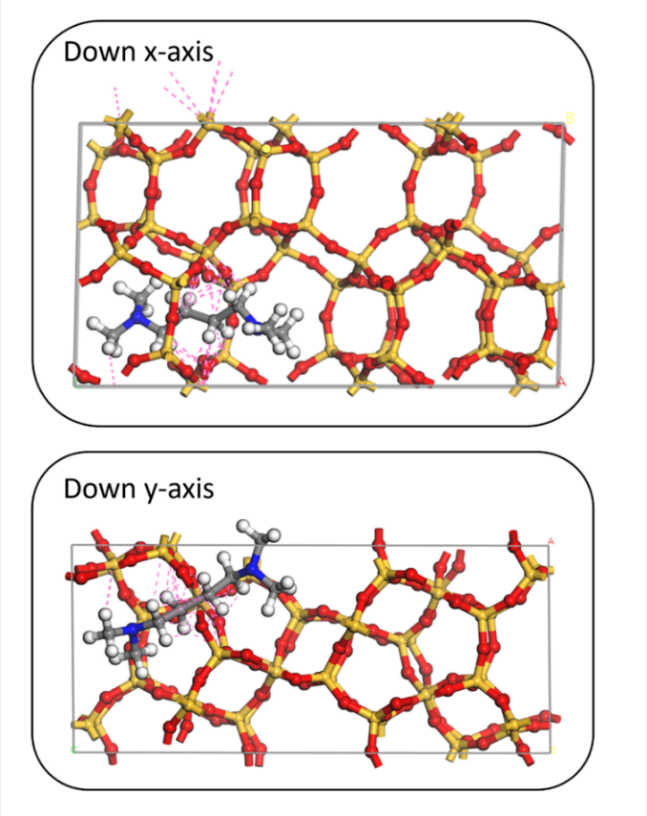


**Figure S8.** Geometry-optimised position of tmdab in pure silica ERS-7, bridging two cages. Close contacts marked with dashed pink dashed lines. This position was simulated with the COMPASS III forcefield in Materials Studio, as per the workflow described in the Experimental Section. The ESV structure was taken from the Atlas of zeolite structures.[1]


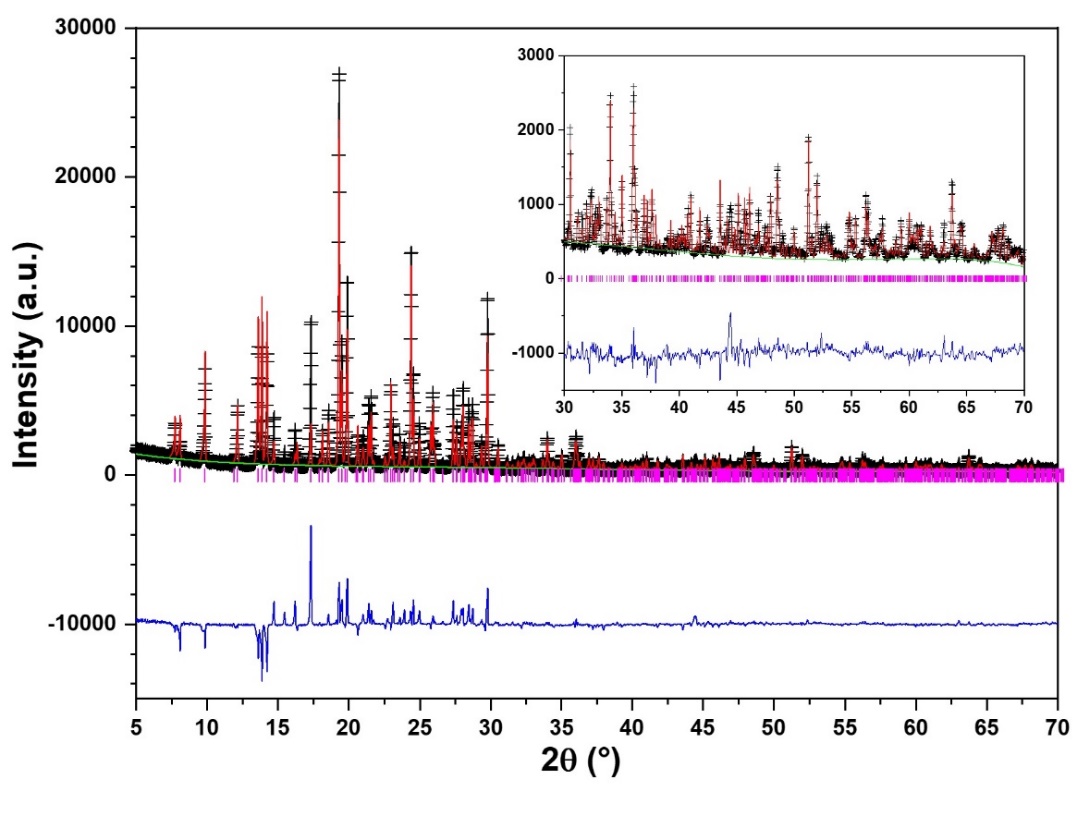


**Figure S9.** Rietveld refinement of ERS-7 modelled with tmdab across 2 cages, as in Fig.S8.


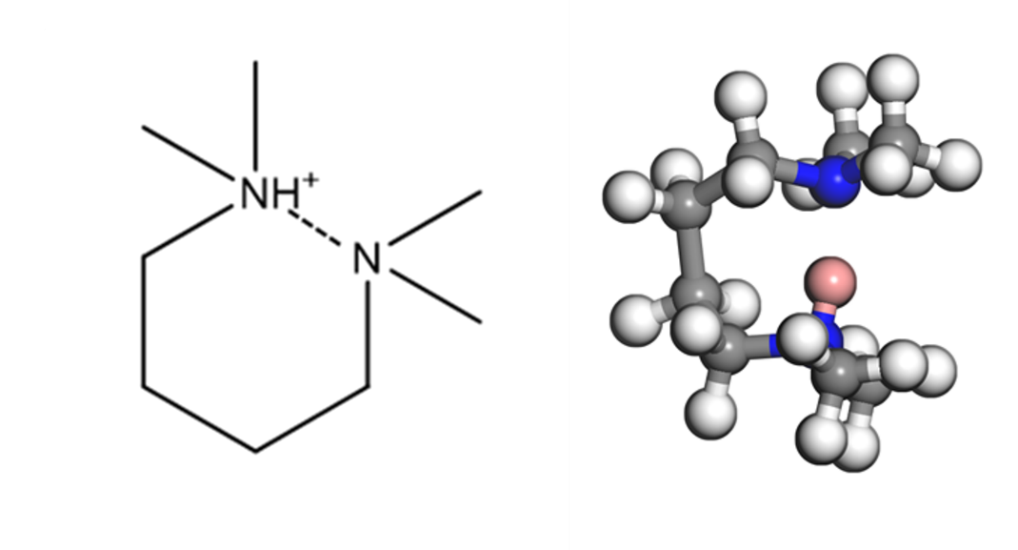


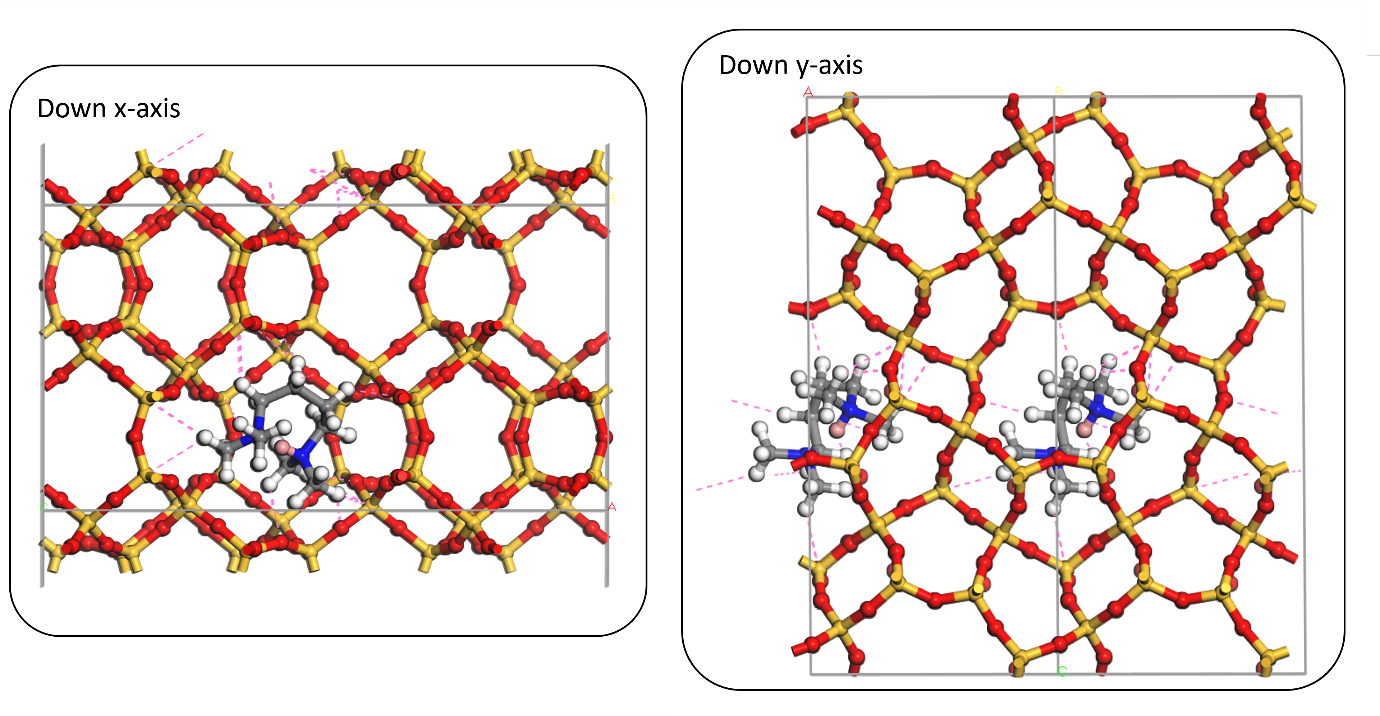


**Figure S10.** (Above) Sketch and geometry-optimised structure of H-bonded 'cyclic' tmdab. The H involved in H-bonding is coloured pink. (Below) Geometry-optimised position of H-bonded cyclic tmdab in ERS-7, shown in an extended view of a unit cell. Close contacts marked with dashed pink lines. This position was simulated with the COMPASS III forcefield in Materials Studio, as per the workflow described in the Experimental Section.

**S4. Low polymer/SiO_2_ : dmpyrr synthesis of mordenite**


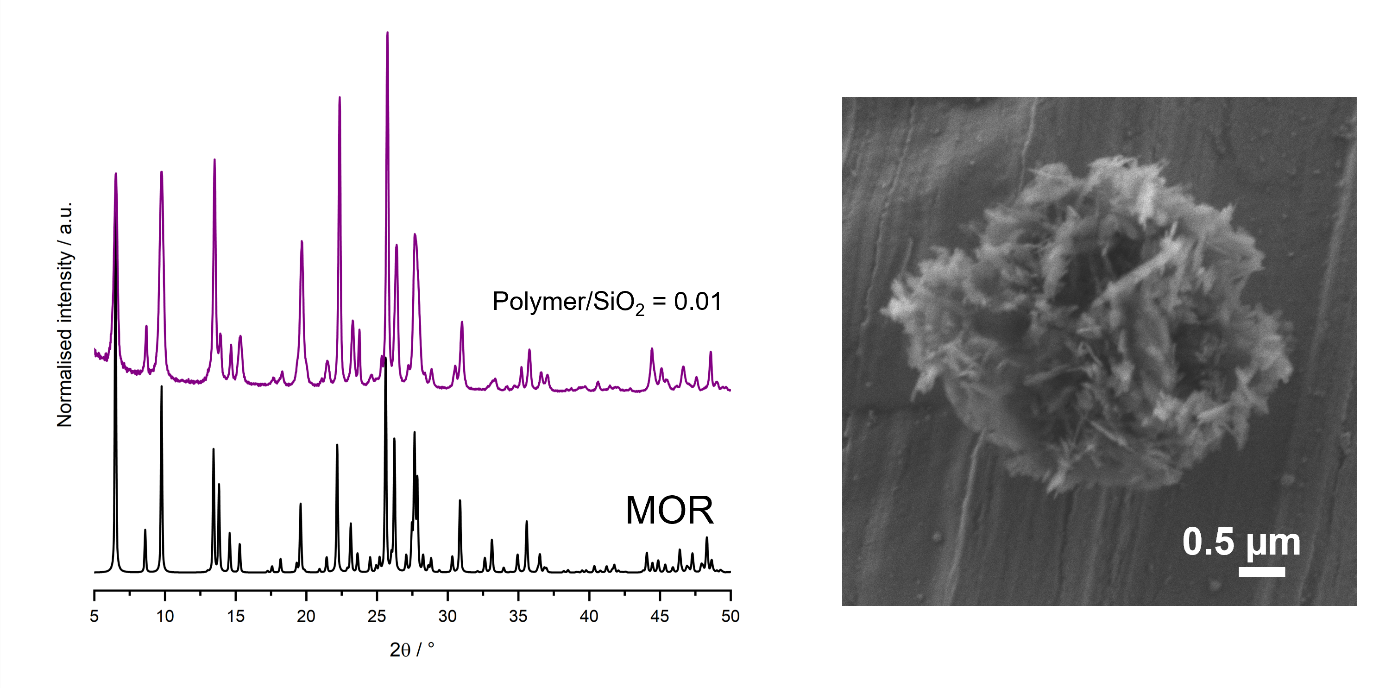


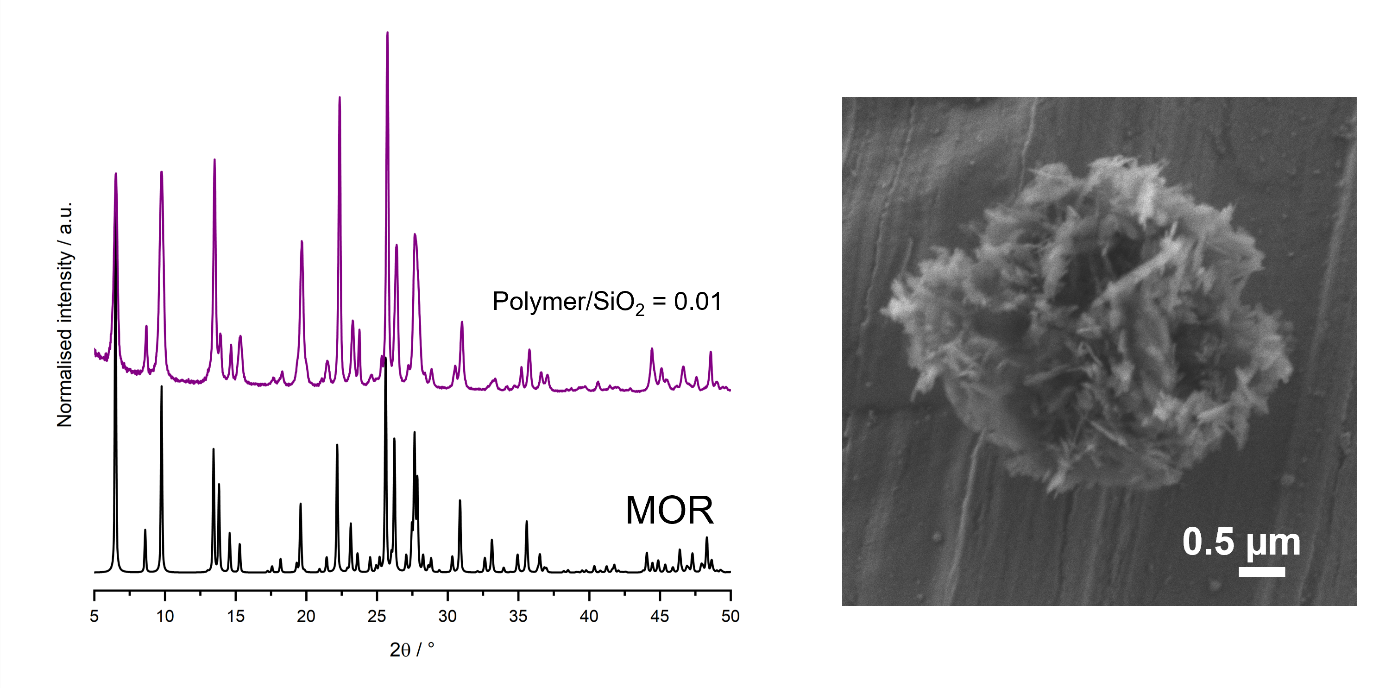


**Figure S11.** (Below) PXRD pattern of zeolite synthesised with very low amounts of polymer in the ERS-7 working synthesis, compared to the simulated pattern of **MOR** topology.[1] (Above) SEM image of the **MOR**-type zeolite synthesised.

**S5. Crystallography of ERS-7 prepared using tmdab and 1,4-dibromobutane**

**Table S2.** Refinement summary

| **Sample** | **As prepared, hydrated ERS-7** | **Calcined, dehydrated Na-ERS-7** |
| --- | --- | --- |
| **Measured chemical formula** | Na_1.1_(C_6_H_14_N)_4_[Si_44.7_Al_3.3_O_96_].3H_2_O | Na_3.2_[Si_44.7_Al_3.2_O_96_] |
| **Refined chemical formula** | Na_1.0_(C_6_H_14_N)_4_[Si_48_O_96_].O_1.5_ | Na_2.0_[Si_48_O_96_] |
| **X-ray source** | Cu Kα | Mo Kα |
| **λ (Å)** | 1.540596 Å | 0.711 Å |
| **T (K)** | 298 K | 298 |
| **Space group** | *Pnma* | *Pnma* |
| ***a* (Å)** | 9.785(1) | 9.803(1) |
| ***b* (Å)** | 12.464(1) | 12.458(1) |
| ***c* (Å)** | 22.946(1) | 22.979(1) |
| **V (Å^3^)** | 2799(1) | 2806(1) |
| ***R*_p_** | 7.2% | 5.3% |
| ***R*_wp_** | 9.4% | 7.4% |
| **χ^2^** | 8.2 | 55 |

**Table S3.** Structure details of calcined Na-ERS-7

| **Site** | **Type** | **x** | **y** | **z** | **Occ.** | **Ω** | **B_iso_** |
| --- | --- | --- | --- | --- | --- | --- | --- |
| T1 | Si | 0.467(2) | 0.876(2) | 0.305(1) | 1 | 8 | 1 |
| T2 | Si | 0.645(2) | 0.378(2) | 0.468(1) | 1 | 8 | 1 |
| T3 | Si | 0.617(2) | 0.507(2) | 0.583(1) | 1 | 8 | 1 |
| T4 | Si | 0.269(2) | 0.012(2) | 0.738(1) | 1 | 8 | 1 |
| T5 | Si | 0.339(2) | 0.874(2) | 0.846(1) | 1 | 8 | 1 |
| T6 | Si | 0.150(2) | 0.877(2) | 0.632(1) | 1 | 8 | 1 |
| O1 | O | 0.333(4) | 0.559(3) | 0.472(2) | 1 | 8 | 1 |
| O2 | O | 0.459(5) | 0.75 | 0.312(2) | 1 | 4 | 1 |
| O3 | O | 0.633(5) | 0.25 | 0.481(2) | 1 | 4 | 1 |
| O4 | O | 0.777(4) | 0.401(3) | 0.429(2) | 1 | 8 | 1 |
| O5 | O | 0.511(4) | 0.418(3) | 0.438(1) | 1 | 8 | 1 |
| O6 | O | 0.175(5) | 0.75 | 0.647(2) | 1 | 4 | 1 |
| O7 | O | 0.062(3) | 0.430(3) | 0.867(2) | 1 | 8 | 1 |
| O8 | O | 0.358(4) | 0.918(3) | 0.259(2) | 1 | 8 | 1 |
| O9 | O | 0.116(3) | 0.903(3) | 0.219(1) | 1 | 8 | 1 |
| O10 | O | 0.325(4) | 0.936(3) | 0.786(2) | 1 | 8 | 1 |
| O11 | O | 0.493(3) | 0.900(2) | 0.873(2) | 1 | 8 | 1 |
| O12 | O | 0.233(3) | 0.918(3) | 0.894(2) | 1 | 8 | 1 |
| O13 | O | 0.323(5) | 0.75 | 0.840(2) | 1 | 4 | 1 |
| O14 | O | 0.279(3) | 0.447(2) | 0.181(2) | 1 | 8 | 1 |
| Na1 | Na | 0.21(2) | 0.75 | 0.75(1) | 0.13(3) | 4 | 1 |
| Na2 | Na | 0.30(2) | 0.75 | 0.379(7) | 0.21(4) | 4 | 1 |
| Na3 | Na | 0.60(2) | 0.75 | 0.602(8) | 0.16(3) | 4 | 1 |

**Table S4.** Crystallographic details of as-prepared ERS-7 samples

| **Site** | **Type** | **x** | **y** | **z** | **Occ.** | **Ω** | **B_iso_** |
| --- | --- | --- | --- | --- | --- | --- | --- |
| T1 | Si | 0.465(1) | 0.873(1) | 0.312(1) | 1 | 8 | 1 |
| T2 | Si | 0.644(1) | 0.372(1) | 0.471(1) | 1 | 8 | 1 |
| T3 | Si | 0.616(1) | 0.512(1) | 0.581(1) | 1 | 8 | 1 |
| T4 | Si | 0.276(1) | 0.017(1) | 0.740(1) | 1 | 8 | 1 |
| T5 | Si | 0.347(1) | 0.873(1) | 0.847(1) | 1 | 8 | 1 |
| T6 | Si | 0.171(1) | 0.873(1) | 0.637(1) | 1 | 8 | 1 |
| O1 | O | 0.339(2) | 0.555(1) | 0.474(1) | 1 | 8 | 1 |
| O2 | O | 0.452(2) | 0.75 | 0.323(1) | 1 | 4 | 1 |
| O3 | O | 0.645(2) | 0.25 | 0.487(1) | 1 | 4 | 1 |
| O4 | O | 0.759(2) | 0.401(1) | 0.424(1) | 1 | 8 | 1 |
| O5 | O | 0.497(2) | 0.400(1) | 0.439(1) | 1 | 8 | 1 |
| O6 | O | 0.198(2) | 0.75 | 0.653(1) | 1 | 4 | 1 |
| O7 | O | 0.057(2) | 0.438(1) | 0.870(1) | 1 | 8 | 1 |
| O8 | O | 0.344(2) | 0.908(1) | 0.266(1) | 1 | 8 | 1 |
| O9 | O | 0.107(2) | 0.902(1) | 0.217(1) | 1 | 8 | 1 |
| O10 | O | 0.334(2) | 0.939(1) | 0.788(1) | 1 | 8 | 1 |
| O11 | O | 0.504(2) | 0.889(1) | 0.873(1) | 1 | 8 | 1 |
| O12 | O | 0.246(2) | 0.920(1) | 0.895(1) | 1 | 8 | 1 |
| O13 | O | 0.324(2) | 0.75 | 0.838(1) | 1 | 4 | 1 |
| O14 | O | 0.281(2) | 0.451(1) | 0.186(1) | 1 | 8 | 1 |
| C1 | C | 0.722(2) | 0.712(6) | 0.447(1) | 0.5 | 8 | 1 |
| C1a | C | 0.948(5) | 0.654(3) | 0.481(1) | 0.5 | 8 | 1 |
| C2 | C | 0.911(2) | 0.698(3) | 0.376(1) | 0.5 | 8 | 1 |
| C3 | C | 0.051(3) | 0.750(8) | 0.365(1) | 0.5 | 8 | 1 |
| C4 | C | 0.050(9) | 0.850(7) | 0.405(1) | 0.5 | 8 | 1 |
| C5 | C | 0.918(8) | 0.839(2) | 0.442(1) | 0.5 | 8 | 1 |
| H1a | H | 0.688(6) | 0.630(8) | 0.439(1) | 0.5 | 8 | 1 |
| H1aa | H | 0.059(5) | 0.660(8) | 0.477(1) | 0.5 | 8 | 1 |
| H1ab | H | 0.923(5) | 0.674(3) | 0.525(1) | 0.5 | 8 | 1 |
| H1ac | H | 0.923(11) | 0.569(2) | 0.474(1) | 0.5 | 8 | 1 |
| H1b | H | 0.661(3) | 0.764(9) | 0.419(1) | 0.5 | 8 | 1 |
| H1c | H | 0.694(2) | 0.732(8) | 0.492(1) | 0.5 | 8 | 1 |
| H2a | H | 0.913(7) | 0.612(3) | 0.367(1) | 0.5 | 8 | 1 |
| H2b | H | 0.839(3) | 0.732(2) | 0.344(1) | 0.5 | 8 | 1 |
| H3a | H | 0.132(2) | 0.695(1) | 0.378(1) | 0.5 | 8 | 1 |
| H3b | H | 0.067(6) | 0.770(9) | 0.319(1) | 0.5 | 8 | 1 |
| H4a | H | 0.140(9) | 0.851(1) | 0.433(1) | 0.5 | 8 | 1 |
| H4b | H | 0.049(1) | 0.923(8) | 0.379(1) | 0.5 | 8 | 1 |
| H5a | H | 0.935(9) | 0.866(3) | 0.486(1) | 0.5 | 8 | 1 |
| H5b | H | 0.843(11) | 0.894(2) | 0.424(1) | 0.5 | 8 | 1 |
| N1 | N | 0.873(1) | 0.724(1) | 0.438(1) | 0.5 | 8 | 1 |
| Na1 | Na | 0.289(6) | 0.25 | 0.249(3) | 0.25 | 4 | 2 |
| Ow1 | O | 0.031(6) | 0.25 | 0.209(2) | 0.38(2) | 4 | 2 |

*R*_wp_ = 7.5%

**Refinement details**

Inspection of some of the T-O-T bond angles in the refined structures show some possess very high values (*ca*. 175°). This may indicate disorder for these sites, or else show that the symmetry used (*Pnma*) does not sufficiently describe the crystal structure. Examination of daughter space groups did not significantly improve the fit, or mitigate the potential issue of the large bridging angle, with the exception of *P*2_1_/*n*11 (equivalent to *P*2_1_/*c* with an alternative axes description). This did improve the T-O-T angles to some extent but did not significantly improve the fit (*R*_wp_ = 8.8%, down from 9.4%) at the expense of a loss of symmetry. Note that the *P*2_1_/*n*11 space group is monoclinic but refinement showed the structure retained a pseudo-orthorhombic setting in this symmetry. We believe that in light of this, materials are best described in *Pnma* but we note the potential for O site disorder and provide details of the alternate *P*2_1_/*n*11 setting for interest.


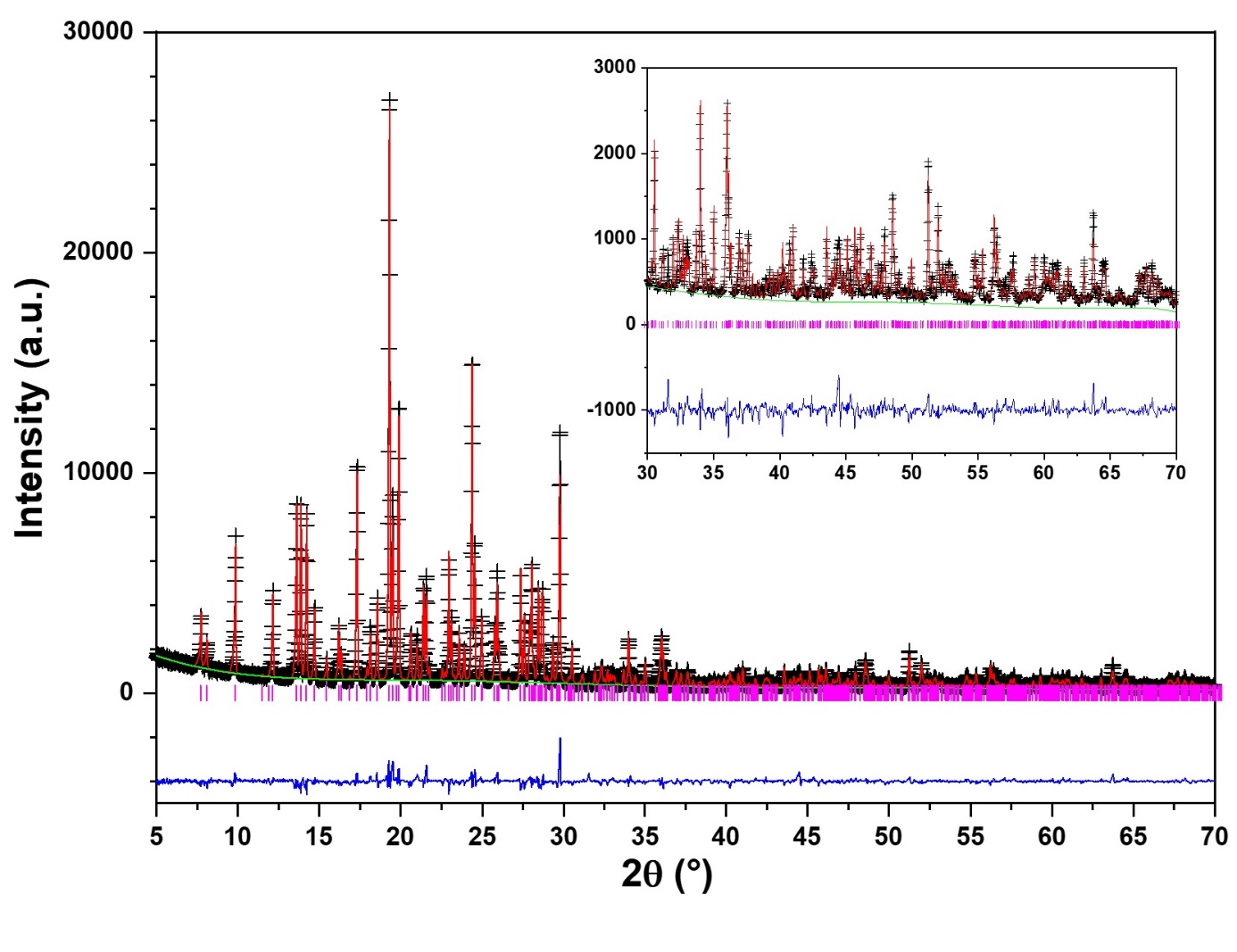


**Figure S12.** Rietveld plot of hydrated, as-prepared ERS-7 in alternative space group *P*2_1_/*n*11.

**Table S5.**

Comparison of refinements in alternate space groups for as-prepared, hydrated ERS-7

| **Space Group** | ***Pnma*** | ***P*2_1_*/n*11 (*P*2_1_/*c*)** |
| --- | --- | --- |
| ***a* (Å)** | 9.785(1) | 9.785(1) |
| ***b* (Å)** | 12.464(1) | 12.464(1) |
| ***c* (Å)** | 22.946(1) | 22.946(1) |
| **V (Å^3^)** | 2799(1) | 2799(1) |
| ***R*_p_** | 7.2% | 6.6% |
| ***R*_wp_** | 9.4% | 8.8% |
| **χ^2^** | 8.2 | 7.2 |

**Table S6**. Crystallographic details of as-prepared ERS-7 samples in alternative *P*2_1_/*n*11 symmetry.

| **Site** | **Type** | **x** | **y** | **z** | **Occ.** | **Ω** | **B_iso_** |
| --- | --- | --- | --- | --- | --- | --- | --- |
| T1 | Si | 0.331(2) | 0.889(2) | 0.865(1) | 1 | 4 | 1 |
| T2 | Si | 0.169(2) | 0.140(2) | 0.361(1) | 1 | 4 | 1 |
| T3 | Si | 0.641(3) | 0.863(2) | 0.532(1) | 1 | 4 | 1 |
| T4 | Si | 0.854(3) | 0.129(2) | 0.027(1) | 1 | 4 | 1 |
| T5 | Si | 0.882(3) | 0.499(2) | 0.918(1) | 1 | 4 | 1 |
| T6 | Si | 0.611(3) | 0.479(2) | 0.419(1) | 1 | 4 | 1 |
| T7 | Si | 0.154(3) | 0.875(2) | 0.655(1) | 1 | 4 | 1 |
| T8 | Si | 0.348(3) | 0.121(2) | 0.155(1) | 1 | 4 | 1 |
| T9 | Si | 0.965(3) | 0.122(2) | 0.812(1) | 1 | 4 | 1 |
| T10 | Si | 0.536(3) | 0.871(2) | 0.311(1) | 1 | 4 | 1 |
| T11 | Si | 0.223(3) | 0.483(2) | 0.760(1) | 1 | 4 | 1 |
| T12 | Si | 0.273(3) | 0.525(2) | 0.255(1) | 1 | 4 | 1 |
| O1 | O | 0.301(2) | 0.767(4) | 0.853(1) | 1 | 4 | 1 |
| O2 | O | 0.177(2) | 0.753(5) | 0.654(1) | 1 | 4 | 1 |
| O3 | O | 0.164(5) | 0.571(3) | 0.718(2) | 1 | 4 | 1 |
| O4 | O | 0.328(4) | 0.427(3) | 0.218(2) | 1 | 4 | 1 |
| O5 | O | 0.965(2) | 0.249(5) | 0.826(1) | 1 | 4 | 1 |
| O6 | O | 0.325(4) | 0.394(3) | 0.726(1) | 1 | 4 | 1 |
| O7 | O | 0.138(4) | 0.608(3) | 0.246(2) | 1 | 4 | 1 |
| O8 | O | 0.112(5) | 0.399(3) | 0.785(2) | 1 | 4 | 1 |
| O9 | O | 0.402(4) | 0.610(3) | 0.277(2) | 1 | 4 | 1 |
| O10 | O | 0.254(5) | 0.916(4) | 0.926(2) | 1 | 4 | 1 |
| O11 | O | 0.250(4) | 0.097(4) | 0.422(2) | 1 | 4 | 1 |
| O12 | O | 0.503(5) | 0.903(3) | 0.879(2) | 1 | 4 | 1 |
| O13 | O | 0.006(4) | 0.111(3) | 0.372(2) | 1 | 4 | 1 |
| O14 | O | 0.287(4) | 0.975(3) | 0.818(2) | 1 | 4 | 1 |
| O15 | O | 0.233(4) | 0.070(2) | 0.308(2) | 1 | 4 | 1 |
| O16 | O | 0.643(2) | 0.750(5) | 0.507(9) | 1 | 4 | 1 |
| O17 | O | 0.656(4) | 0.941(3) | 0.476(2) | 1 | 4 | 1 |
| O18 | O | 0.832(4) | 0.040(3) | 0.977(2) | 1 | 4 | 1 |
| O19 | O | 0.484(4) | 0.910(3) | 0.557(1) | 1 | 4 | 1 |
| O20 | O | 0.992(4) | 0.106(3) | 0.068(2) | 1 | 4 | 1 |
| O21 | O | 0.962(3) | 0.449(3) | 0.864(2) | 1 | 4 | 1 |
| O22 | O | 0.575(3) | 0.581(3) | 0.378(2) | 1 | 4 | 1 |
| O23 | O | 0.753(5) | 0.575(4) | 0.900(2) | 1 | 4 | 1 |
| O24 | O | 0.738(5) | 0.421(4) | 0.394(2) | 1 | 4 | 1 |
| C1 | C | 0.057(7) | 0.118(4) | 0.595(1) | 1 | 4 | 1 |
| C1a | C | 0.017(6) | 0.299(3) | 0.634(1) | 1 | 4 | 1 |
| C2 | C | 0.979(4) | 0.261(2) | 0.528(1) | 1 | 4 | 1 |
| N1 | N | 0.972(1) | 0.217(1) | 0.589(1) | 1 | 4 | 1 |
| C3 | C | 0.854(9) | 0.334(7) | 0.520(1) | 1 | 4 | 1 |
| C4 | C | 0.745(5) | 0.283(1) | 0.560(1) | 1 | 4 | 1 |
| C5 | C | 0.822(2) | 0.194(7) | 0.594(1) | 1 | 4 | 1 |
| H1aa | H | 0.957(12) | 0.373(2) | 0.633(1) | 1 | 4 | 1 |
| H1ab | H | 0.008(5) | 0.268(3) | 0.678(1) | 1 | 4 | 1 |
| H1ac | H | 0.123(8) | 0.323(8) | 0.627(1) | 1 | 4 | 1 |
| H1a | H | 0.164(6) | 0.134(9) | 0.586(1) | 1 | 4 | 1 |
| H1b | H | 0.026(12) | 0.054(3) | 0.566(1) | 1 | 4 | 1 |
| H1c | H | 0.051(10) | 0.085(5) | 0.639(1) | 1 | 4 | 1 |
| H2a | H | 0.074(7) | 0.305(4) | 0.519(1) | 1 | 4 | 1 |
| H2b | H | 0.973(3) | 0.199(2) | 0.495(1) | 1 | 4 | 1 |
| H3a | H | 0.877(15) | 0.415(5) | 0.535(1) | 1 | 4 | 1 |
| H3b | H | 0.821(9) | 0.340(9) | 0.475(1) | 1 | 4 | 1 |
| H4a | H | 0.702(9) | 0.342(12) | 0.590(2) | 1 | 4 | 1 |
| H4b | H | 0.661(3) | 0.250(15) | 0.534(2) | 1 | 4 | 1 |
| H5a | H | 0.786(3) | 0.189(8) | 0.639(1) | 1 | 4 | 1 |
| H5b | H | 0.794(8) | 0.117(8) | 0.574(1) | 1 | 4 | 1 |
| Na1 | Na | 0.248(6) | 0.75 | 0.754(3) | 0.25 | 4 | 2 |
| Ow1 | O | 0.010(5) | 0.75 | 0.788(2) | 0.47(2) | 4 | 2 |

**S6. Modelling of dmpip and dmpyrr within ERS**

**Table S7.**

| **Cation** | **Combined energy / kcal mol^–1^** | **Zeolite Energy / kcal mol^1^** | **Binding energy / kcal mol^–1^** |
| --- | --- | --- | --- |
| Dimethylpiperidinium (dmpip) | –38562.4 | –26022.1 | –555.9 |
| Dimethylpyrrolidinium (dmpyrr) | –37012.0 | –26025.6 | –555.0 |

**S7. Oligomers of cationic polymer 1 energy minimised within ESV and MOR**

Modelling results show that when cationic polymer 1 adopts the ‘zig-zag’ conformation observed in the formation of **ITH**,^[2]^ this can be accommodated inside the **ESV** framework by bridging across the cages via the 8R windows. Although the polymer is able to fit inside the **ESV** framework with this ‘zig zag’ conformation, the binding energy is relatively low (–140.52 kcal/mol) given the number of cages that the molecule spans across. It seems that the polymer is unlikely to exert a strong ‘templating’ effect for the **ESV** structure but is unlikely to hinder its synthesis. In contrast, the **MOR** framework is not able to accommodate the polymer in this conformation (Figure S13). This becomes apparent when trying to dock the molecule ‘by eye’. Wherever the molecule is placed, there are significant numbers of bad contacts between polymer and the framework. This suggests that the polymer is not likely to fit inside the framework without a significant change in its conformation. This is confirmed by energy minimising the polymer inside the **MOR** framework, which leads to a significantly more linear conformation of the oligomer.


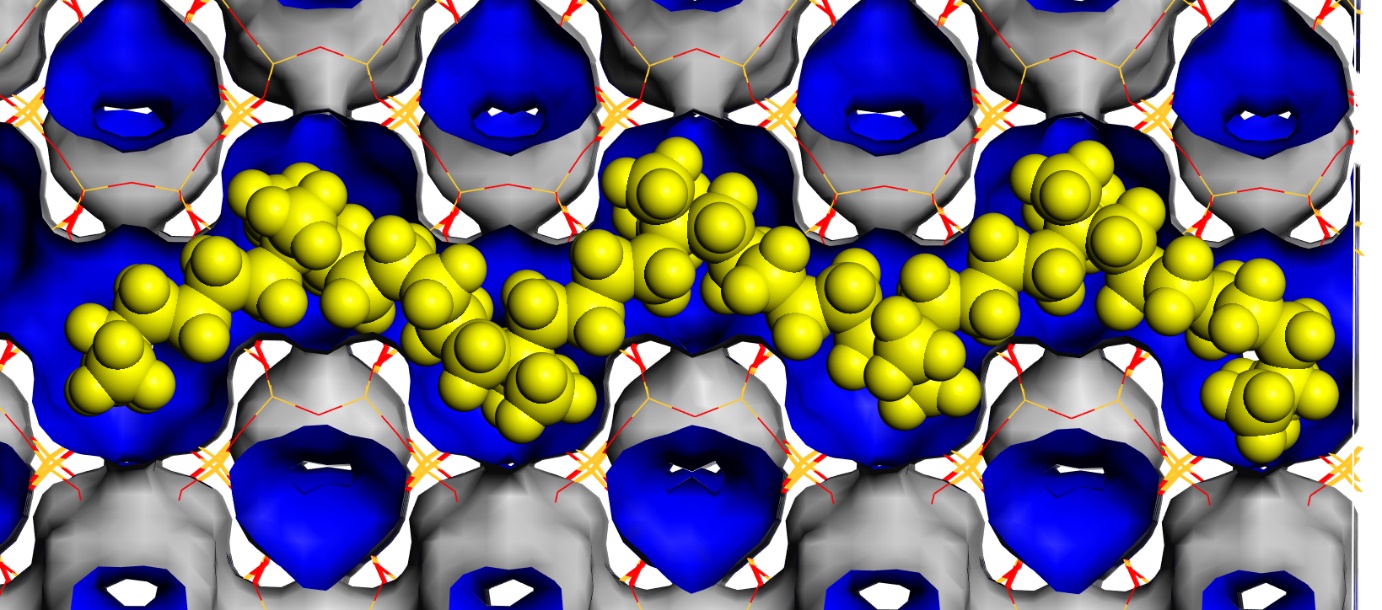


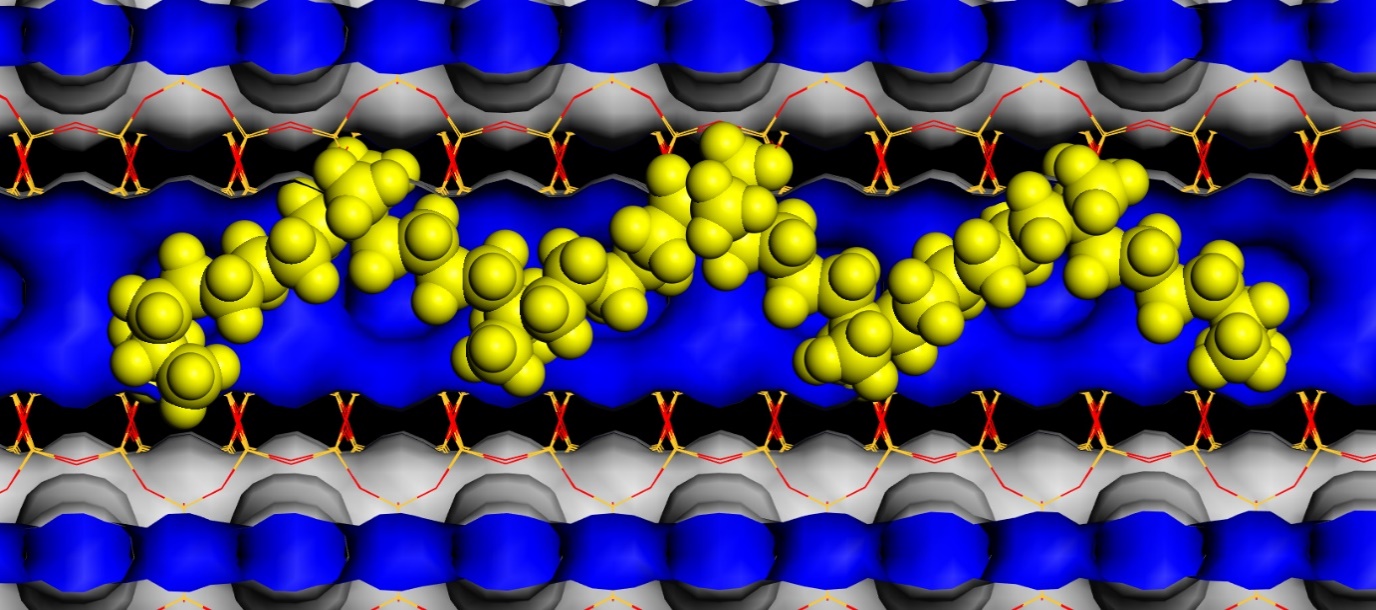


**Figure S13.** (above) The energy minimised location for an oligomer of polymer **1** inside the ESV framework. The oligomer (yellow) fits within the channel system of the framework. The inside of the channel system is shown in blue. (Below) Polymer **1** in the ‘zig-zag’ conformation docked ‘by eye’ in the channel system of the **MOR** framework. Note that the oligomer (yellow) does not fit wholly within the channel system. The inside of the channel system is shown in blue. The MOR structure was obtained from the IZA database.^[1]^ This position was simulated with the COMPASS III forcefield in Materials Studio, as per the workflow described in the Experimental Section.

**S8. References**

[1] IZA database, <http://www.iza-structure.org/databases/>

[2] C. Lei, Z. Dong, C. Martínez, J. Martínez‐Triguero, W. Chen, Q. Wu, X. Meng, A. Parvulescu, T. De Baerdemaeker, U. Müller, A. Zheng, Y. Ma, W. Zhang, T. Yokoi, B. Marler, D. E. De Vos, U. Kolb, A. Corma, F. Xiao, *Angew. Chem. Int. Ed.*, 2020, **59**, 15649–15655.
